# Meta-Regression on the Heterogenous Factors Contributing to the Prevalence of Mental Health Symptoms During the COVID-19 Crisis Among Healthcare Workers

**DOI:** 10.3389/fpsyt.2022.833865

**Published:** 2022-03-18

**Authors:** Xi Chen, Jiyao Chen, Meimei Zhang, Rebecca Kechen Dong, Jizhen Li, Zhe Dong, Yingying Ye, Lingyao Tong, Ruiying Zhao, Wenrui Cao, Peikai Li, Stephen X. Zhang

**Affiliations:** ^1^Chinese Open Science Network, Xiamen, China; ^2^Department of Strategy and Entrepreneurship, College of Business, Oregon State University, Corvallis, OR, United States; ^3^Department of Speech-Language-Hearing Sciences, Texas Tech University Health Sciences Center, Lubbock, TX, United States; ^4^UniSA Business, University of South Australia, Adelaide, SA, Australia; ^5^Department of Innovation, Entrepreneurship and Strategy, School of Economic and Management, Tsinghua University, Beijing, China; ^6^Department of Sociology and Interuniversity Center for Social Science Theory and Methodology (ICS), University of Groningen, Groningen, Netherlands; ^7^Department of Psychology and Behavioral Sciences, Zhejiang University, Hangzhou, China; ^8^Department of Clinical, Neuro- and Developmental Psychology, Vrije Universiteit Amsterdam, Amsterdam, Netherlands; ^9^Department of Social, Health and Organizational Psychology, Utrecht University, Utrecht, Netherlands; ^10^Entrepreneurship, Innovation and Family Enterprise Discipline, Adelaide Business School, University of Adelaide, Adelaide, SA, Australia

**Keywords:** meta-regression, systematic review, meta-analysis, COVID-19, mental health, healthcare workers, frontline healthcare workers

## Abstract

**Objective:**

This paper used meta-regression to analyze the heterogenous factors contributing to the prevalence rate of mental health symptoms of the general and frontline healthcare workers (HCWs) in China under the COVID-19 crisis.

**Method:**

We systematically searched PubMed, Embase, Web of Science, and Medrxiv and pooled data using random-effects meta-analyses to estimate the prevalence rates, and ran meta-regression to tease out the key sources of the heterogeneity.

**Results:**

The meta-regression results uncovered several predictors of the heterogeneity in prevalence rates among published studies, including severity (e.g., above severe vs. above moderate, *p* < 0.01; above moderate vs. above mild, *p* < 0.01), type of mental symptoms (PTSD vs. anxiety, *p* = 0.04), population (frontline vs. general HCWs, *p* < 0.01), sampling location (Wuhan vs. Non-Wuhan, *p* = 0.04), and study quality (*p* = 0.04).

**Conclusion:**

The meta-regression findings provide evidence on the factors contributing to the prevalence rate of mental health symptoms of the general and frontline healthcare workers (HCWs) to guide future research and evidence-based medicine in several specific directions.

**Systematic Review Registration:**

https://www.crd.york.ac.uk/prospero/display_record.php?RecordID=220592, identifier: CRD42020220592.

## Introduction

Since the first publicly known cases in Wuhan, China, on November 17, 2019, the COVID-19 (coronavirus disease 2019) crisis has become one of the worst epidemics in human record (1). The sudden outburst of this highly infectious disease and the containment measures such as quarantine and social distancing have posed immense pressure on the work and life of the healthcare workers (HCWs) (2–4). During the COVID-19 pandemic, HCWs have to face increased workload and extended working hours, shortage of medical resources, risk of nosocomial infection, stigmatization and other related problems (5–7). These work-related issues may induce the emotional distress of HCWs to cause mental health symptoms such as anxiety, depression, burnout, or sleep issues (8). Frontline HCWs are in a unique position to suffer mentally in particular. They have to deal with the ethical dilemma of resources allocation and the work pressure of hospice care (5, 9, 10) while being exposed to a high risk of infection in COVID-infected wards. The infection or death of any family member or colleague could act as additional stressors resulting mental health problems (11, 12).

Several early (rapid) meta-analysis papers have appeared but they pooled HCW of different countries all together. However, such practices inadvertently contribute to the differences in their prevalence rates, given the large heterogeneity in terms of not only the COVID cases and deaths but also the containment strategies and hospital capacities and readiness to handle COVID-19 cases across countries (13, 14). To rule out such heterogeneity at the same time, we conducted meta-regression analysis by focusing on a single country, China, which has had a sufficient number of empirical studies to analyze several factors at the same time to better understand the heterogenous factors contributing to the prevalence rate of mental health symptoms of the general and frontline healthcare workers (HCWs) (15, 16). Such evidence on the heterogenous factors contributing to the prevalence rate of mental health symptoms provide directions to better guide this important and proliferating stream of research.

## Methods

This meta-regression analysis with a systematic review and meta-analysis conducted in accordance with the Preferred Reporting Items for Systematic Reviews and Meta-Analyses (PRISMA) statement 2019 and registered in the International Prospective Register of Systematic Reviews (PROSPERO: CRD42020220592).

### Data Sources and Search Strategy

**Figure 1:**
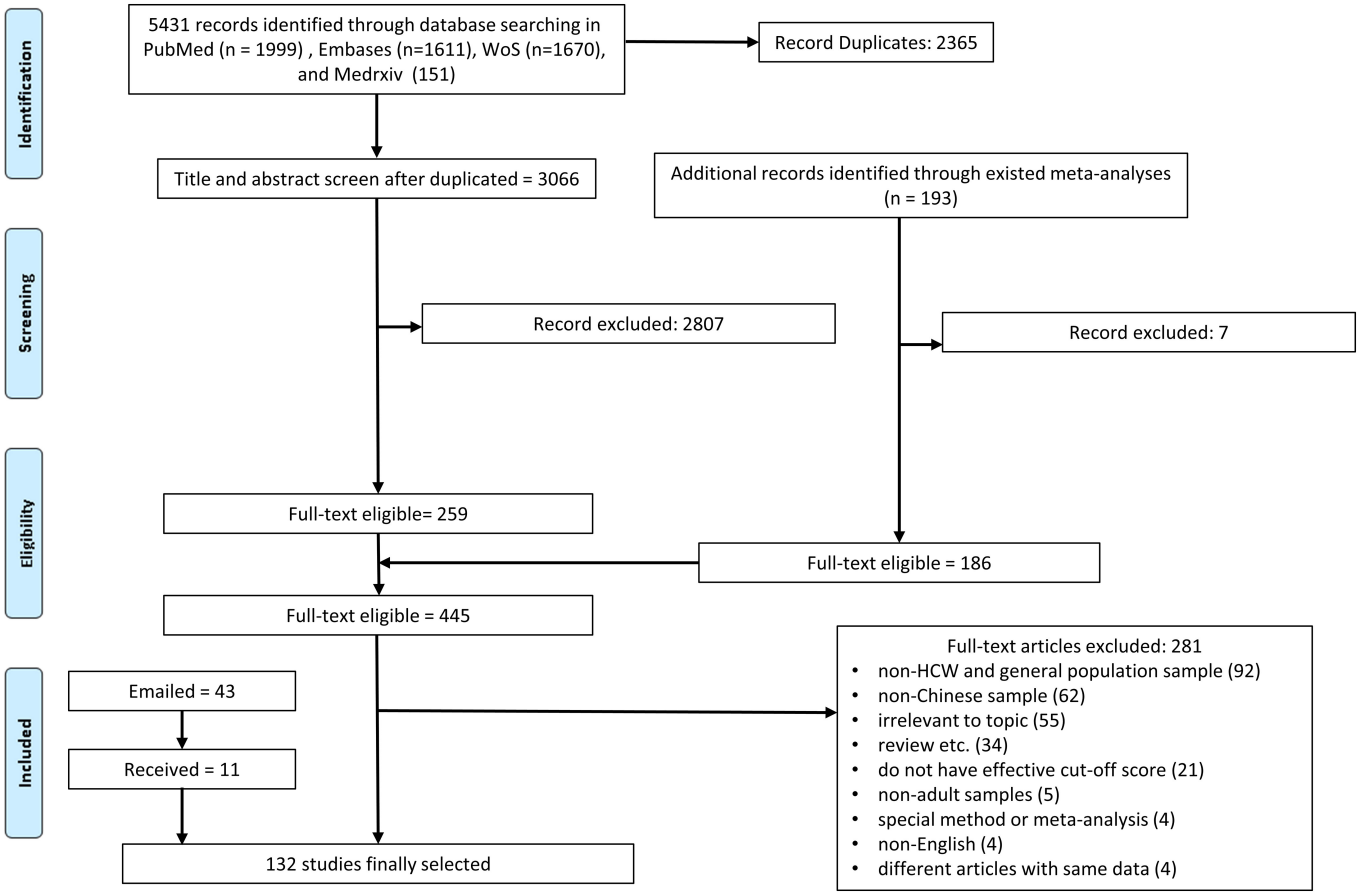
The PRISMA (Preferred Reporting Items for Systematic Reviews and Meta-Analysis) flow diagram.

We conducted a comprehensive literature search in the databases of *PubMed, Embase*, and *Web of Science*. Our search query, shown in Supplementary Table S1, was entered with Boolean operators to search the titles, abstracts, keywords, and subject headings (for example, Mesh terms) in each database. To account for preprints, we searched *medRxiv* (medrxiv.org). We started our search on November 10, 2020, and finalized it on November 16, 2020, in order to cover the first year when HCWs faced a crisis of the COVID-19, as after 1 year the number of COVID-19 cases dropped significantly in China to no longer pose a crisis situation for HCWs (17). Figure 1 details the flow chart of our search process.

### Selection Criteria

The studies are included in our meta-analysis based on the following criteria:

- a. Context: COVID-19 crisis in China.
- b. Population: frontline HCWs, general HCWs, and general adult population (for comparison).
- c. Outcome: at least one mental symptom outcomes, e.g., anxiety, depression, distress, insomnia, and PTSD.
- d. Instrument: validated scales with cutoff points for the mental health outcomes.
- e. Language: English.

According, we excluded studies that meet the following criteria:

- a. Population: children, adolescents, or specific niche adult populations such as COVID-19 patients, inpatients, or other patients, adults under quarantine, pregnant/postpartum women.
- b. Methodological approaches: Non-primary studies such as reviews or meta-analyses, qualitative or case studies without a validated instrument, interventional studies, interviews, or news reports.
- c. Measurements: Non-validated mental health instruments (i.e., self-made questionnaire) or instruments without a validated cutoff score to calculate a prevalence rate (i.e., STAI, SCL-90 for anxiety and depression).

We contacted the authors of papers that missed some critical information if the articles:

1. a. Contain primary data on mental health of relevant population using established instruments under COVID-19 period but do not report the prevalence rates. For example, a study may report the mean and SD of our outcomes but not their prevalence rates.
2. b. Surveyed a sample that mixed our targeted population and other populations, such as children, in a manner such that we could not extract the prevalence rate(s) for our targeted population. We included the studies that authors provided prevalence rate for our targeted population only and excluded the studies with mixed populations.
3. c. Miss some critical information, such as the data collection time or location.
4. d. Are unclear on critical information. For example, some articles are unclear whether they used the cutoff for above mild or above moderate symptoms to calculate the overall prevalence rates of mental health symptoms. When a cutoff point is reported for an overall prevalence, above mild or above moderate was assigned based on the typical cutoff point of that instrument.

### Selection Process and Data Extraction

The articles that passed the inclusion criteria were exported into an EndNote library where we identified duplications and then imported to Rayyan for screening. Two researchers (L.T. and Y.Y.) independently screened the articles based on their titles and abstracts. If both coders excluded an article independently, it was excluded.

Six researchers (X.C, M.Z., R.C., Z.D., R.D., B.C.) were paired to assess the eligibility of each paper based on reading its full text and extracting the relevant data into a coding book based on a coding protocol. The coding book records information such as the authors and year of the paper, title, publication status, sample locations, date of data collection, sample size, response rate, population, age (mean, SD, min and max), gender proportion, instruments, cutoff scores used, the prevalence/mean/SD of the mental health outcome, and other notes or comments. Pairs of researchers first double-coded and crosschecked each paper independently. The remaining discrepancies after the crosscheck were discussed between the pair of coders. In cases where a pair of coders continued to disagree, a lead coder (X.C.) checked the paper independently and discussed it with the two original coders to determine its coding. The lead coder also integrated and reviewed all the coding information. Particularly, the lead coder checked the mental outcomes, instruments, outcome levels, and cutoff scores reported given the multitude of reporting practices in individual papers. We were able to identify papers that used unusual cutoff scores later for sensitivity analysis.

### Assessment of Bias Risk

Following other meta-analyses (18, 19), we used the Mixed Methods Appraisal Tool (MMAT) (20), including seven questions to conduct the quality assessment of the studies. Pairs of coders independently evaluated the risk of bias and quality of the studies and rated them based on the MMAT. Most discrepancies were resolved through a discussion between the pair of researchers, and any disagreement after discussions was resolved by a lead researcher. Papers were classed into high (6–7) or medium quality (lower than 6).

### Statistical Analysis

To analyze the data in a consistent manner, we ensure the independence of mental health symptoms and samples. For instance, for studies that examine a mental health outcome with more than one instrument, we report the results based on the most popular instrument. If a study reported several prevalence rates by several cutoffs, we use one of them, in the following order of preference: above severe, above moderate, and above mild. Thus, only one prevalence rate for a mental health outcome in a sample is entered to ensure the samples remain independent.

The overall prevalence and 95% confidence intervals of psychological outcomes were pooled using Stata 16.1. Similar to prior studies on the prevalence of mental symptoms, the random-effects model was used to extract the pooled estimates (21). We reported the heterogeneity by the *I*^2^ statistic, which measures the percentage of variance resulting from true differences in the effect sizes rather than the sampling error (22). We performed subgroup analyses by the key potential sources of heterogeneity of outcomes (five types of mental health symptoms), severity of outcome (above mild/above moderate/above severe), three major population groups (frontline HCWs, general HCWs, and general population for comparison), and instrument type for each outcome. Furthermore, given the high degree of heterogeneity of the true differences in the effect sizes, we ran a meta-regression to regress the prevalence upon not only these three category variables (outcome, severity, and population) but also female proportion, data collection time, data collection location (Wuhan vs. Non-Wuhan), sample size, and study quality. We included data collection time to examine whether the mental symptoms change over time dynamically. While the COVID-19 crisis continues to evolve, there is a lack of dynamic analysis on the mental symptoms of any population over time. Sensitivity analysis was conducted, and Funnel plots were used to assess publication bias. Significance level was set as two-sided and *p* < 0.05.

## Results

### Study Screening

Our systematic search (Figure 1) across all the databases yielded 5,431 potentially relevant papers, out of which 2,365 were duplications and removed. Of the remaining 3,066 papers, we screened their titles and abstracts in the first stage and the full text of the 445 articles in the second stage. We also emailed the authors of 43 articles that missed critical information and were able to get the information to include 11 additional studies. Altogether, the process generated 132 articles.

### Study Characteristics

The 132 papers included contains 171 samples (Supplementary Table S2) with a total of 645,805 individual participants. Table 1 summarizes their key characteristics. Among the 171 independent samples, about a quarter of them studied frontline HCWs and general HCWs (27.5 and 26.2%, respectively), and almost half studied the general population (43.3%) as a comparison. More than one-third of samples covered anxiety and depression. Another one-third investigated other mental symptoms including insomnia, PTSD, and distress, (15.9, 9.3, and 3.0%, respectively). Respectively, 20.7, 41.6, and 32.0% of samples reported prevalence rates at the mild above, moderate above, and severe above level by the severity of the symptoms.

**Table 1:** Characteristics of the studies on mental health in China in a year of COVID-19 epidemic.

**Characteristics**	**Total number of studies/samples**	**Percent**	**Level of analysis**
*Population*			Sample
Frontline HCWs	47	27.5	
General HCWs	50	26.2	
General population (for comparison)	74	43.3	
*Outcome*			Prevalence
Anxiety	123	36.8	
Depression	117	35.0	
Distress	10	3.0	
Insomnia	53	15.9	
PTSD	31	9.3	
*Severity*			Prevalence
Above mild	69	20.7	
Above moderate	139	41.6	
Above severe	107	32.0	
Overall	19	5.67	
*Sampling location*			Article
Wuhan	35	20.5	
Non-Wuhan	136	79.5	
*Sampling date*			Article
January 2020	9	6.1	
February 2020	90	65.9	
March 2020	23	17.4	
April 2020	9	6.8	
May 2020	1	0.6	
June 2020	2	2.3	
July 2020	2	0.8	
*Design*			Article
Cross-sectional	128	97.0	
Cohort	4	3.0	
*Publication status*			Article
Preprint	10	7.6	
Accepted	1	0.8	
Published	121	91.7	
*Quality*			Article
Good	92	77.3	
Medium	30	22.7	
	**Median**	**Range**	
Number of participants	742	30–123,768	Article
Female portion	69%	12–100%	Article
Response rate	85%	14–100%	Article

Almost all the studies, 126 out of 131, employed cross-sectional surveys; specifically, 9 (6.1%) conducted the survey in January 2020, 90 (65.9%) in February, 23 (17.4%) in March, and 14 (10.6%) in April or later. Almost one-quarter of them (20.5%) contained a sample targeting populations in Wuhan. Most studies were published in journals, and 10 (7.6%) studies remained as preprints. The assessment based on the Mixed Methods Appraisal Tool (MMAT) indicated 100 (77.3%) studies were of good quality (score no <6 out of 7) and 31 studies were of medium quality (score <6 but >4). The median number of individuals per sample was 742 (range: 30–123,768) with a median female proportion of 69% (range: 12–100%) and a median response rate of 85% (range: 14–100%).

The 131 papers employed a wide arrange of instruments to assess mental health (Supplementary Table S3). GAD (61.8%) and SAS (23.6%) are the first and second most popular measures for anxiety, and PHQ (65.0%) and SDS (14.5%) for depression; distress is measured the most by K6 (50.0%); insomnia is measured by ISI (66.0%) and PSQI (26.4%); and PTSD by IES-R (41.9%), PCL-C (25.8%), and PCL-5 (25.8%). Please see the details in Supplementary Table S3.

### Pooled Prevalence Rates of Mental Health Symptoms

The prevalence rates of the 171 samples were pooled by the subgroups one at a time (Table 2). First, the overall prevalence rates of mental health symptoms that surpassed the cutoff values of mild, moderate, and severe were 30, 15, and 2%, respectively. The overall prevalence of mental health symptom frontline HCWs and general HCWs are 16 and 13%, respectively, and in comparison, the prevalence in the general population is 13%. The overall prevalence of anxiety, depression, distress, insomnia, and PTSD are 11, 14, 15, 17, and 21%. Figure 2 graphically depicts such findings of the pooled analysis by subgroups using forest plots.

**Table 2:** The pooled prevalence rates of mental health symptoms by subgroups of population, outcome, and severity.

**First-level subgroup**	**Second-level subgroup**	Number of samples (*K*)^*^	**Percent (%)**	**Sample size (*N*)**	**Prevalence (%)**	**95% CI**	***P*** **value**
Population	Frontline HCWs	47	27.5	66,208	16	13–19	<0.001
	General HCWs	50	29.2	92,357	13	10–16	<0.001
	General population (for comparison)	74	43.2	487,240	13	11–15	<0.001
Outcome^#^	Anxiety	123	36.8	306,102	11	9–13	<0.001
	Depression	117	35.0	157,254	14	11–17	<0.001
	Distress	10	3.0	71,675	15	8–25	<0.001
	Insomnia	53	15.9	87,426	17	13–21	<0.001
	PTSD	31	9.3	23,348	21	12–32	<0.001
Severity^#^	Above mild	69	20.7	52,448	30	27–33	<0.001
	Above moderate	139	41.6	242,030	15	14–16	<0.001
	Above severe	107	32.0	323,777	2	2–3	<0.001
	Overall	19	5.67	27,550	30	27–33	<0.001

*CI, Confidence Interval*.

* *The total independent samples are larger than the number of studies because some studies included multiple samples*.

# *The total sample sizes are larger than the total sample of the 171 independent samples because one sample can assess multiple mental health outcomes*.

**Figure 2:**
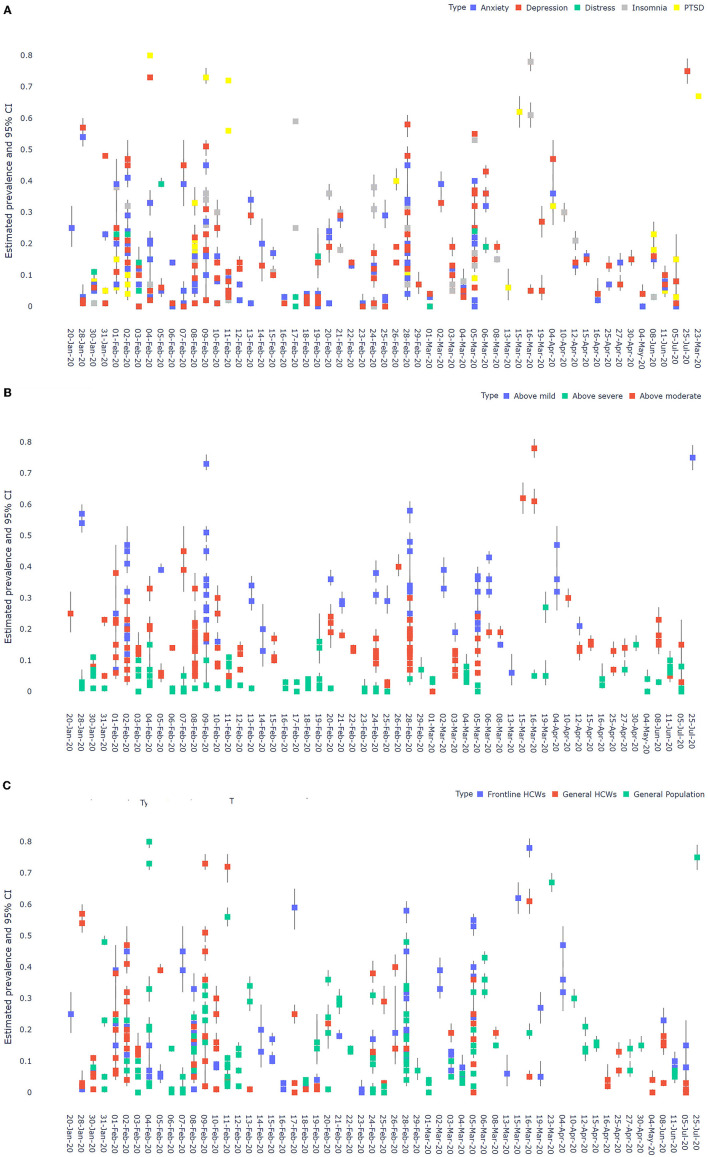
**(A)** A forest plot of the pooled prevalence by outcomes. **(B)** A forest plot of the pooled prevalence by outcome levels. **(C)** A forest plot of the pooled prevalence by population.

### Meta-Regression on the Prevalence of Mental Health Symptoms

As pooled sub-group analysis takes account of only one factor at a time, to better explain the heterogeneity of the prevalence of mental health symptoms, Table 3 reports the results of a meta-regression analysis that takes account of several factors at the same time. The meta-analytical model explained over 40% of the variance of mental health symptoms among these studies (*R-squared* = 56.8%, *tau*^2^ = 0.09).

**Table 3:** The results of meta-regression of mental health symptoms during COVID-19.

**Variables**	**Coefficient (CI, 95%)**	**Std. Err**.	***P*** **-value**
**Outcome**			
Anxiety (reference)			
Depression	0.07 (−0.00 to 0.14)	0.04	0.064
Distress	0.03 (−0.16 to 0.21)	0.10	0.817
Insomnia	0.06 (−0.04 to 0.15)	0.05	0.251
PTSD	0.13^*^ (0.01 to 0.25)	0.06	0.039
**Severity**			
Above mild	−0.30^**^ (−0.45 to −0.15)	0.08	<0.001
Above moderate	−0.64^***^ (−0.78 to −0.50)	0.07	<0.001
Above severe	−1.05^***^ (−1.20 to −0.90)	0.08	<0.001
Overall (reference)			
**Population**			
Frontline HCWs	0.12^**^ (0.03 to 0.20)	0.04	0.005
General HCWs (reference)			
General population	0.08 (−0.01 to 0.17)	0.04	0.078
**Publication Status**			
Preprint (reference)			
Accepted	−0.23 (−0.65 to 0.18)	0.21	0.265
Published	−0.06 (−0.20 to 0.07)	0.07	0.338
*Female proportion*	0.15 (−0.09 to 0.39)	0.12	0.233
*Date of data collection*	0.00 (0.00 to 0.00)	0.00	0.392
*Wuhan vs. Non-Wuhan sample*	−0.09^*^ (−0.17 to −0.00)	0.04	0.038
*Sample size*	0.00 (0.00 to 0.00)	0.00	0.124
*Quality*	0.07^*^ (0.01 to 0.13)	0.03	0.036
Constant	−8.01	10.4	0.438
R^2^	0.56		
Wald X^2^ (16)	419.18^***^		<0.001

* *p < 0.05*,

** *p < 0.01*,

*** *p < 0.001*.

The prevalence of severe mental health symptoms is significantly lower than that of moderate mental illness (*p* < 0.001), which is in turn significantly lower than those of mild mental illness (*p* < 0.001). The prevalence of mental health symptoms of frontline HCWs is significantly higher than that of general HCWs (*p* = 0.005). General HCWs and the general population do not differ in their mental health prevalence rates. The prevalence rates of PTSD (*p* = 0.039) is significantly higher than that of anxiety. Interestingly, the prevalence of mental health symptoms of participants in Wuhan, the epicenter of the COVID-19 crisis in China, was significantly lower than that in Non-Wuhan samples (*p* = 0.038). The prevalence rates of mental health symptoms were higher in studies of papers with a higher quality rating (*p* = 0.036). The female proportion (*p* = 0.233), date of data collection (*p* = 0.392), sample size of studies (*p* = 0.124), or publication status (*p* = 0.265) did not predict the prevalence rates significantly.

The meta-analytical regression results enable the prediction of prevalence rates while taking account of the influence of multiple factors and hence offer a superior model over the earlier pooled analyses. In other words, the meta-regression model considers multiple predictors of mental health symptoms in a single model at the same time instead of the approach of considering one predictor at a time by pooled prevalence, the typical method to estimate the prevalence of mental health symptom in prior meta-analytical papers in COVID-19 literature.

Hence, based on the results of the meta-regression, we report the predicted prevalence rates of varying severity levels of the different mental health symptoms of frontline HCWs, general HCWs, and the general population. Table 4 show the predicted prevalence rates of mental health symptoms by populations, outcomes, and severity by the meta-analytical regression model. The prevalence rates vary greatly by the mental health outcomes and severity. The prevalence rates are lower when using a higher level of severity, which drives the heterogeneity of prevalence rate to a large degree. Among the different types of mental health outcomes, distress seems to be the most prevalent among all three populations.

**Table 4:** The predicted prevalence rates of mental health symptoms by populations, outcomes, and severity by the meta-analytical regression model.

	**Prevalence rate (95% CI)**		
**Mental health symptoms above certain severity**	**Frontline HCWs**	**General HCWs**	**General population** **(for comparison)**
Above mild anxiety	0.29 (0.24–0.33)	0.23 (0.19–0.28)	0.27 (0.23–0.31)
Above moderate anxiety	0.15 (0.12–0.18)	0.11 (0.08–0.14)	0.13 (0.11–0.16)
Above severe anxiety	0.04 (0.02–0.05)	0.02 (0.01–0.03)	0.03 (0.02–0.04)
Above mild depression	0.32 (0.27–0.36)	0.27 (0.22–0.31)	0.3 (0.26–0.35)
Above moderate depression	0.17 (0.14–0.21)	0.13 (0.1–0.16)	0.16 (0.13–0.19)
Above severe depression	0.05 (0.03–0.07)	0.03 (0.02–0.04)	0.04 (0.03–0.06)
Above mild distress	0.3 (0.21–0.39)	0.24 (0.16–0.33)	0.28 (0.19–0.37)
Above moderate distress	0.16 (0.09–0.23)	0.12 (0.06–0.18)	0.14 (0.08–0.21)
Above severe distress	0.04 (0.01–0.09)	0.02 (0–0.06)	0.03 (0.01–0.08)
Above mild insomnia	0.31 (0.26–0.37)	0.26 (0.21–0.31)	0.29 (0.24–0.35)
Above moderate insomnia	0.17 (0.13–0.21)	0.13 (0.1–0.16)	0.15 (0.12–0.19)
Above severe insomnia	0.05 (0.03–0.07)	0.03 (0.01–0.05)	0.04 (0.02–0.06)
Above mild PTSD	0.34 (0.28–0.41)	0.29 (0.23–0.36)	0.33 (0.26–0.39)
Above moderate PTSD	0.2 (0.15–0.24)	0.15 (0.11–0.2)	0.18 (0.14–0.23)
Above severe PTSD	0.06 (0.03–0.1)	0.04 (0.02–0.07)	0.05 (0.03–0.09)

*CI, Confidence Interval*.

### Sensitivity Analysis

**Figure 3:**
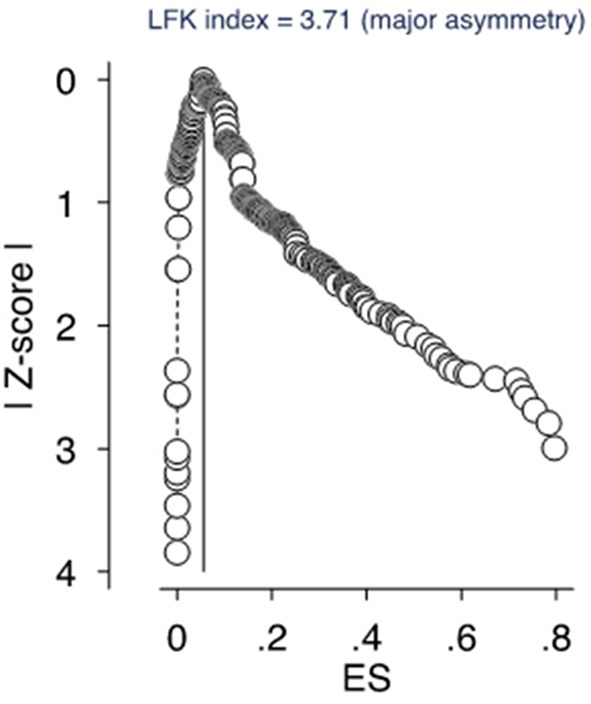
The DOI plot and the Luis Furuya–Kanamori (LFK) index.

Our meta-analytical regression model was able to take account of the impact of several factors, such as publication status (insignificant), sample size (insignificant), and article quality score (significant). Furthermore, we conducted our analysis with the exclusion of each study one-by-one from the meta-analytic model and found it did not significantly alter the findings. The visual inspection of the sensitivity plot however revealed that there is significant asymmetry. Figure 3 reports the DOI plot in combination with the Luis-Kanamori (LFK) index, which has higher sensitivity and power than a funnel plot (23, 24). An LFK index scores of ±1, between ±1 and ±2, or ±2 indicating “no asymmetry”, “minor asymmetry”, and “major asymmetry”, respectively, and hence the LFK index of 3.7 represents major asymmetry. Therefore, the presence of publication bias is likely.

## Discussion

Our meta-regression analysis from a systematic review comprises 171 independent samples with 645,805 participants from 132 studies, at least an order of magnitude larger than the prior meta-analyses that included 7–50 studies with 2,123–62,382 participants (15, 16, 25–27). Moreover, prior meta-analyses examined the prevalence rates of mental health symptoms based on one level of the severity of symptoms (i.e., above mild), and we included articles that reported the prevalence at varying levels of severity of symptoms. Our meta-regression results based on multiple factors are consistent yet fine-tune the previous results, a comparison reveals that our pooled prevalence rates largely fall between the findings of previous meta-analyses, suggesting. For example, our findings show similar prevalence of depression (32 vs. 32%) but a higher prevalence of anxiety (29 vs. 24%) for frontline HCWs reported by Bareeqa et al. (15). Similarly, our findings show similar prevalence of anxiety for the general HCWs (23 vs. 23%), but a higher prevalence of depression (27 vs. 23%) in Pappa et al. (16). The two differences between our prevalence rates and the prior reports are statistically significant given the large sample size involved, and hence we significantly update the cumulative evidence on mental health prevalence rates in COVID-19.

### Meta-Regression Findings

We were able to conduct meta-regression to account for the influence of multiple predictors at the same time to enable better prediction on the prevalence of each mental health symptom thanks to the large number of samples in China over a year of the COVID-19 crisis. The meta-regression evidence shows that several predictors are significantly associated with prevalence rates of mental symptoms during COVID-19, including the population, the severity and type of mental symptoms, sampling location, and study quality.

Frontline HCWs suffered more than general HCWs and the general population did across all five types of mental symptoms. It is also worth noting the general HCWs did not significantly differ from general populations across any mental symptoms. Such a result implies that whether a HCW is frontline could be a major factor in shaping her/his mental health, because of the risk of more direct exposure to the COVID crisis situation. In other words, the fact that general HCWs work in the medical field alone may not trigger much mental health symptoms than the general population has. Hence, our evidence suggests that policymakers need to prioritize frontline HCWs in particular in this ongoing pandemic. We call upon healthcare organizations to test specific psychological support intervention programs as well as mental health prevention plans to help HCWs (28).

The severity of mental symptoms, which has been unaccounted for in prior meta-analyses, was found to contribute greatly to the heterogeneity of prevalence rates, hence individual mental health papers need to pay special attention to the severity with clarity. Otherwise, researchers and practitioners might mix the severity of severe, moderate, and mild mental illness. Since prior meta-analyses largely examined the prevalence rates of mild mental health symptoms, yet psychiatrists care not only the mild symptoms, and the significant differences revealed by this study call for more meta-regression analyses on varying levels of severity to provide evidence for practitioners relevant to their concerns.

Among the five mental health symptoms examined, PTSD had the highest prevalence rates in both general and frontline HCWs. Our findings suggest that practitioners need to be aware and pay more attention to PTSD under the COVID-19 pandemic. Moreover, given that more than three-quarters of existing empirical studies focused on anxiety and depression, we call out for future research to focus on mental PTSD.

Past mental health research has reported inconsistent results on the relationship between individuals' mental symptoms and their locations. Some studies reported that mental symptoms increase along with the distance to the epicenter in the COVID-19 pandemic, known as “typhoon eye effect” (29–31). However, other findings have demonstrated an opposite effect, where mental symptoms decrease as the distance to the epicenter increases, known as the “ripple effect” (32, 33). Our accumulative evidence shows that people in the epicenter of China in Wuhan suffered less mental symptoms than those outside of Wuhan, lending support to the typhoon eye effect. This finding suggests future research to differentiate, report, and possibly model sampling locations based on the epicenter of a pandemic to enable better geographical identification of mental symptoms (34–36).

Our findings that the samples in papers with higher quality tend to find higher prevalent rates of mental symptoms suggest study quality may matter. Particularly, future meta-analysis may pay attention to the representativeness of sampling, the response rate, etc., to better account for the heterogeneity in the pooled prevalence rates.

As the COVID-19 epidemic evolves, we expected the mental symptoms may change over time. However, the evidence of meta-regression using time as a predictor failed to reveal significant effect, and a potential reason might be the development of COVID-19 in various parts of China happened at varying paces, and more refined studies are needed to uncover the change of prevalence rates effect over time across COVID waves (37).

### Study Limitations and Future Research

This research has a few limitations. First, the validity of our findings rests upon the quality and reporting of the original studies. While we paid extra attention to the severity, the cutoff points, and the ways in which individual articles used this information, the multitude of varying practices contributes to additional noise and variance in the analysis. Second, since we included studies in English, which may result in some biases. Third, 97.9% of the primary studies included were cross-sectional surveys, and we call for more cohort studies to examine the effect of time. Fourth, we examine the major adult population of interest, and future research could examine other populations that could be vulnerable, such as hospitality workers, professional athletes, and managers (38–41). As research on COVID pandemic continues to develop, future research may also explore other factors, such as age, health conditions, COVID testing availability, and conspiracy belief in COVID (42–44).

Finally, we only focus on studies that collected data in one country (China) to reduce the heterogeneity of different situations across countries, and we call for future meta-analyses in other countries or regions where data are sufficient -see meta-analyses on several regions including Africa, Eastern Europe, Latin America, South Asia, Southeast Asia, and Spain (45–50).

## Conclusion

This meta-regression analysis takes account of several heterogeneities to analyze the evidence on the prevalence rates of mental health symptoms of healthcare workers under the COVID-19 crisis to provide a foundation of the past research and to guide future effort. Our findings suggest further research and practices on mental health symptoms need to better specify and account for the heterogeneous factors identified as such heterogeneity contribute to significant differences of the prevalence of mental health symptoms reported.

## Author Contributions

XC: investigation, data curation, visualization, writing—original draft, writing—review and editing, and project administration. JC: conceptualization, methodology, validation, formal analysis, investigation, resources, data curation, visualization, writing—original draft, writing—review and editing, and supervision. JL: investigation, writing—review and editing, and resources. MZ, RD, ZD, YY, and LT: investigation (Data). RZ, WC, and PL: investigation. SZ: conceptualization, methodology, validation, formal analysis, investigation, data curation, writing—original draft, writing—review and editing, and supervision. XC, JC, and SZ: co-lead this project. All authors were involved in approving the manuscript. All authors contributed to the article and approved the submitted version.

## Funding

This work was supported by the National Natural Science Foundation of China (71772103).

## Conflict of Interest

The authors declare that the research was conducted in the absence of any commercial or financial relationships that could be construed as a potential conflict of interest.

## Publisher's Note

All claims expressed in this article are solely those of the authors and do not necessarily represent those of their affiliated organizations, or those of the publisher, the editors and the reviewers. Any product that may be evaluated in this article, or claim that may be made by its manufacturer, is not guaranteed or endorsed by the publisher.
