# Supplementary material for: Meta-Regression on the Heterogenous Factors Contributing to the Prevalence of Mental Health Symptoms During the COVID-19 Crisis Among Healthcare Workers
Source: Front Psychiatry. 2022 Mar 18;13:833865. doi: 10.3389/fpsyt.2022.833865 (PMC8972157; doi:10.3389/fpsyt.2022.833865)
Supplement: Supplementary file 1 [file Data_Sheet_1.pdf]

Supplementary Material

Table S1: The search string used in this systematic review and meta-analysis
(Nov 17, 2019 - Nov 16, 2020)

Search	Search topic	Search keywords (titles, abstracts, and subject headings) with Boolean operators
1	Exposure/ Context	"coronavirus" OR "SARS-COV-2" OR "COVID-19" OR “2019nCoV” OR “2019-nCoV”
2	Outcome of interest	"depressi*" OR "anxi*" OR "insomnia" OR "sleep disorder" OR "sleep issue" OR "sleep problem" OR "PTSD" OR "distress disorder" OR "distress symptom" OR "distress issue" OR "distress problem" OR "mental disorder" OR "mental issue" OR "mental problem" OR "psychiatric disorder" OR "psychiatric issue" OR "psychiatric problem"
3	Country	China
4	Language	English
Overall search		1 AND 2 AND 3

Table S2. The characteristics of the individual studies included (1-132)

Authors, year	Population	Sample size	Wuhan vs. Not	Female proportion	Outcome (%)					Instrument	Outcome Severity
					ANX	DEP	INS	DIS	PTSD		
An et al., 2020	GHCW	1103	No	90.7	NA	5.3	NA	NA	NA	PHQ-9	Severe
Ben-Ezra et al., 2020	GP	1134	No	53.5	NA	NA	NA	19.1	NA	K-6	Moderate
Cai et al., 2020	FHCW	348, 343	Yes	96.5<br>97.4	16.4<br>4.6	21.8<br>9.2	14.7<br>6.1	NA	33.3<br>19.1	GAD-7, PHQ-9, ISI, IES-R	Moderate
	GHCW	361<br>278			4.7<br>7.6	9.0<br>12.8	9.0<br>13.7	NA	16.9<br>21.3		
Cao et al., 2020	FHCW	37	No	78.4	NA	18.9	NA	NA	NA	PHQ-9	Moderate
Chen et al., 2020a	GHCW	105	No	90.5	1.9	3.8	NA	NA	NA	SAS, SDS	Severe
Chen et al., 2020b	GP	1921	No	NA	0.8	0.0	NA	NA	NA	SAS	Severe
Chen et al., 2020c	GHCW	422	Yes	44.6	NA	3.8	NA	NA	71.6	SDS<br>IES-R	Mild (DEP)<br>Overall (PTSD)
	GP	1071			NA	2.9	NA	NA	55.8		
Chen et al., 2020d	FHCW	95	No	74.5	8.5	8.5	1.1	NA	14.9	GAD-7, PHQ-9, ISI, PCL-C	Moderate (PTSD)<br>Severe (ANX, DEP, & INS)
	GHCW	77		59.7	0.0	1.3	0.0	NA	3.0		
Chen et al., 2020e	GHCW	902	No	68.6	16.6	18.3	NA	NA	NA	GAD-7, PHQ-9	Moderate
Cheng et al., 2020	GHCW	534	No	82.4	0.7	NA	30.0	NA	NA	SAS	Moderate (INS)<br>Severe (ANX)
Choi et al., 2020	GP	500	No	54.8	14.0	7.0	NA	NA	NA	GAD-7, PHQ-9	Moderate (ANX)<br>Severe (DEP)
Dai et al., 2020	GHCW	4357	No	76.8	NA	NA	NA	39.1	NA	GHQ-12	Mild
Dong et al., 2020	FHCW	4618	No	83.7	NA	NA	NA	24.2	NA	HEI	Overall
Du et al., 2020	FHCW	134	Yes	39.4	20.1	12.7	NA	NA	NA	BAI, BDI-II	Mild
Elhai et al., 2020	GP	908	No	82.8	2.6	3.7	NA	NA	NA	GAD-7, DASS-21	Severe
Fang, 2020	GP	191	Yes	60.2	NA	6.8	NA	NA	NA	PHQ-9	Severe
Feng, 2020	GP	671	No	53.9	3.9	12.2	NA	NA	10.4	GAD-7, PHQ-2, PC-PTSD	Mild (PTSD)<br>Moderate (DEP)<br>Severe (ANX)
Fong et al., 2020	GP	590	No	63.2	NA	15.3	NA	NA	NA	PHQ-9,	Severe

Fu et al., 2020	GP	1242	Yes	69.7	27.5	29.3	30.0	NA	NA	GAD-7, PHQ-9<br>AIS	Mild (ANX & DEP)<br>Overall (INS)
Gao et al., 2020	GP	4872	No	67.7	22.6	48.3	NA	NA	NA	GAD-7, WHO-5	Moderate (ANX)<br>Overall (DEP)
Guo et al., 2020a	FHCW	3351	No	74.8	2.2	2.5	NA	NA	NA	SAS, SDS	Severe
	GHCW	7767			0.8	1.2	NA	NA	NA		
Guo et al., 2020b	GP	2441	No	52.5	NA	72.6	20.6	NA	79.6	CES-D-20, PSQI,<br>PCL-5	Overall (DEP &<br>PTSD)<br>Moderate (INS)
Han et al., 2020	FHCW	21199	No	98.6	0.8	1.3	NA	NA	NA	SAS, SDS	Severe
Hong et al., 2020	FHCW	4692	No	96.9	8.1	9.4	NA	NA	NA	GAD-7, PHQ-9	Moderate
Hou et al., 2020	GP	3063	No	56.3	13.3	14.1	NA	NA	NA	GAD-2, PHQ-2	Moderate
Hu et al., 2020a	FHCW	645	No	75.0	39.0	33.0	NA	NA	NA	GAD-7, PHQ-9	Mild
Hu et al., 2020b	FHCW	2014	Yes	87.1	3.3	1.1	NA	NA	NA	SAS, SDS	Severe
Huang et al., 2020a	GP	6261	No	57.3	4.9	8.0	NA	NA	NA	SAS, PHQ-9	Moderate (ANX)<br>Severe (DEP)
Huang et al., 2020b	FHCW	364	No	58.8	0.8	NA	NA	NA	NA	SAS	Severe
Huang et al., 2020c	GP	1172	No	69.3	33.0	NA	24.7	NA	NA	GAD-7, ISI	Mild (ANX)<br>Overall (INS)
Jin et al., 2020	GP	143	Yes	NA	1.4	NA	NA	NA	NA	SAS	Severe
		175	No		0.0	NA	NA	NA	NA		
Juan et al., 2020	FHCW	416	No	70.6	4.6	6.4	NA	NA	NA	GAD-7, PHQ-9	Moderate
Lai et al., 2020	GHCW	1257	No	76.7	5.3	6.2	1.0	10.5	NA	GAD-7, PHQ-9,<br>ISI, IES-R	Severe
Lam et al., 2020	FHCW	932	No	75.2	NA	11.2	NA	NA	NA	PHQ-9	Severe
Lei et al., 2020	GP	1593	No	61.3	0.4	1.1	NA	NA	NA	SAS, SDS	Severe
Leng et al.2020	FHCW	90	Yes	72.2	NA	NA	NA	NA	5.6	PCL-C	Mild
Li et al., 2020a	FHCW	219	Yes	78.1	NA	NA	58.9	NA	NA	SRQ-20, AIS	Overall
	GHCW	729	No	76.4	NA	NA	25.0	NA	NA		
Li et al., 2020b	GHCW	4396	Yes	100.0	25.2	14.2	NA	NA	NA	GAD-7, PHQ-9	Moderate
Li et al., 2020c	GP	3637	No	63.0	16.1<br>27.5	22.7<br>31.2	26.2<br>33.7	NA	NA	GAD-7, PHQ-9<br>ISI	Mild

Li et al., 2020d	GP	1109	No	43.9	NA	NA	NA	NA	67.9	IES-R	Overall
Li et al., 2020e	GHCW	908	No	75.6	0.8	1.2	NA	NA	NA	SAS, SDS	Severe
Li et al., 2020f	GP	88611	No	76.9	13.67	NA	NA	NA	NA	GAD-7	Moderate
Li et al., 2020g	FHCW	176	Yes	77.3	50.0	NA	NA	NA	NA	HAMA	Moderate
Li et al., 2020h	FHCW	225	Yes	72.0	35.6	46.7	NA	NA	31.6	DASS-21, IES-R	Mild (ANX, DEP & PTSD)
Li et al., 2020i	FHCW	356	No	86.2	NA	NA	NA	NA	61.8	PCL-5	Moderate
Li et al., 2020j	FHCW	150	No	62.7	39.3	44.7	NA	NA	NA	HAMA, HAMD	Moderate
Liang et al., 2020	FHCW	899	No	81.3	14.2	24.3	12.4	NA	NA	GAD-7, PHQ-9, ISI	Moderate
	GP	1174		69.5	8.9	17.3	6.7	NA	NA		
Lin et al., 2020a	GHCW	2316	No	NA	41.1	46.9	32.0	NA	NA	GAD-7, PHQ-9, ISI	Mild
Lin et al., 2020b	GP	5461	No	70.1	9.2	10.8	2.7, 1.6	NA	NA	GAD-7, PHQ-9, ISI	Severe
Liu et al., 2020a	GHCW	512	No	84.6	0.8	NA	NA	NA	NA	SAS	Severe
Liu et al., 2020b	GP	14592	No	68.4	6.6	10.1	NA	NA	NA	GAD-7, PHQ-9, SRQ-20	Severe
Liu et al., 2020c	GP	285	Yes	54.4	NA	NA	NA	NA	7.0	PCL-5	Moderate
Liu et al., 2020d	GHCW	4976	No	82.3	2.2	2.3	NA	15.9	NA	SAS, SDS, SRQ-20	Overall (DIS) Severe (ANX & DEP)
Liu et al., 2020e	GP	4991	No	50.4	1.50	NA	NA	NA	NA	SAS	Severe
Liu et al., 2020f	GP	574	No	57.7	NA	0.7	NA	NA	NA	SDS	Severe
Liu et al., 2020g	FHCW	317	No	80.2	12.7	18.9	NA	NA	NA	GAD-7, PHQ-9	Moderate
	GHCW	719			13.6	18.2	NA	NA	NA		
Liu et al., 2020h	FHCW	742	No	85.5	4.0	2.1	NA	NA	NA	DASS-21	Severe
	GHCW	1289			1.2	1.0	NA	NA	NA		
Lu et al., 2020a	GHCW	2042	No	77.6	2.9	0.3	NA	NA	NA	HAMA, HAMD	moderate (ANX) severe (DEP)
	GP	257			1.6	0.0	NA	NA	NA		
Lu et al., 2020b	GHCW	1848	No	63.0	NA	18.8	NA	NA	NA	CES-D-9	Moderate
Lu et al., 2020c	FHCW	382	Yes	61.8	7.6	9.9	NA	NA	6.8	GAD-7, PHQ-9, PCL-C	Moderate (PTSD) Severe (ANX and DEP)
	GP	1035		91.4	6.3	6.8	NA	NA	4.5		
Mi et al., 2020	GHCW	1029	No	61.6	6.6	13.3	NA	NA	NA	GAD-2, HQ-2	Moderate

Ni et al., 2020a	GHCW	214	Yes	68.8	23.8	19.2	NA	NA	NA	GAD-2, PHQ-2	Overall (DEP) Moderate (ANX)
	GP	1577		60.8	22.0	19.2	NA	NA	NA		
Ni et al., 2020b	GP	2551	No	68.9	1.8	14.9	NA	NA	NA	GAD-7, PHQ-2	Moderate (DEP) Severe (ANX)
Ning et al., 2020	GHCW	612	No	72.9	16.3	25.0	NA	NA	NA	SAS, SDS	Mild
Pan et al., 2020a	GHCW	194	Yes	81.8	3.6	5.2	NA	NA	NA	GAD-7,PHQ-9	Moderate
Pan et al., 2020b	GP	3035	No	46.9	NA	5.6	NA	NA	NA	PHQ-9	Moderate
Pan et al., 2020c	GHCW	423	No	89.4	0.5	NA	NA	NA	NA	SAS	Severe
Qi et al., 2020	FHCW	801	No	79.9	NA	NA	78.4	NA	NA	PSQI	Moderate
	GHCW	505		81.2	NA	NA	61.0	NA	NA		
Qian et al., 2020a	GP	510	Yes	50.0	32.7	NA	NA	NA	NA	GAD-7	Moderate
		501	No	49.1	20.4	NA	NA	NA	NA		
Qian et al., 2020b	GP	510	Yes	50.0	32.8	NA	NA	NA	NA	GAD-7	Moderate
		501	No	49.1	20.5	NA	NA	NA	NA		
Qiu et al., 2020	GP	52730	No	64.7	NA	NA	NA	5.1	NA	CPDI	Severe
Que et al., 2020	GHCW	2285	No	69.1	11.6	12.8	6.8	NA	NA	GAD-7, PHQ-9, ISI	Moderate
Ren et al., 2020	GP	6130	No	66.9	7.1	12.0	NA	NA	NA	GAD-7, PHQ-9	Moderate
Shi et al., 2020	FHCW	9725	No	52.1	11.9	12.8	7.0	NA	NA	GAD-7, PHQ-9	Moderate
	GP	46954			10.0	10.4	5.5	NA	NA		
Si et al., 2020	GHCW	863	No	70.7	13.6	13.9	NA	NA	40.2	DASS-21, IES-6	Overall (ANX & DEP) Moderate (PTSD)
Song et al., 2020a	GHCW	14825	No	64.3	NA	25.2	NA	NA	9.1	PCL-5, CES-D-20	Mild (DEP) Moderate (PTSD)
Song et al., 2020b	GP	709	No	74.2	12.7	13.5	20.7	NA	NA	GAD-7, CES-D-10, ISI	Mild (INS), Moderate (ANX & DEP)
Su et al., 2020	GP	403	No	68.5	5.0	NA	NA	NA	NA	GAD-7	Severe
Sun et al., 2020a	GP	2091	No	60.8	NA	NA	NA	NA	4.6	PCL-5	Moderate
Sun et al., 2020b	FHCW	170	Yes	92.4	1.2	0.0	NA	NA	NA	DASS-21	Severe
Sun et al., 2020c	GP	472	No	56.4	NA	75.4	NA	NA	NA	CES-D-10	Mild
Sun et al., 2020d	GHCW	536	No	69.0	9.7	18.8	NA	NA	NA	GAD-7, PHQ-9	Mild

Tan et al., 2020	GP	673	No	25.6	1.3	0.9	0.4	NA	10.8	DASS-21, ISI, IES-R	Moderate (PTSD), Severe (ANX, DEP, & INS)
Teng et al., 2020	GHCW	398	No	75.9	0.3	4.3	NA	NA	NA	SAS, PHQ-9	Severe
Tu et al., 2020	GHCW	100	No	100.0	10.0	2.0	2.0	NA	NA	GAD-7, PHQ-9, PSQI	Severe
Wang et al., 2020a	GP	600	No	55.5	0.0	0.3	NA	NA	NA	SAS, SDS	Severe
Wang et al., 2020b	GHCW	123	Yes	90.0	7.3	25.2	38.2	NA	NA	SAS, SDS, PSQI	Mild (ANX & DEP) Moderate (INS)
Wang et al., 2020c	GHCW	1045	No	85.8	20.0	13.6	10.4	NA	NA	HADS, ISI	Moderate
Wang et al., 2020d	GP	6437	No	56.1	NA	NA	17.7	NA	NA	PSQI	Moderate
Wang et al., 2020e	FHCW	274	No	77.4	NA	NA	19.7	NA	NA	PSQI	Mild
Wang et al., 2020f	FHCW	179	No	52.0	14.5	5.3	7.3	NA	NA	GAD-7, PHQ-9, ISI	Severe
	GP	16066			2.7	2.0	3.1	NA	NA		
Wang et al., 2020g	FHCW	661	No	64.5	24.8	36.6	23.6	NA	NA	HADS, PSQI	Mild (ANX & DEP) Moderate (INS)
	GHCW	853			22.3	36.6	17.0	NA	NA		
	GP	487			20.1	31.8	13.3	NA	NA		
Wang et al., 2020h	FHCW	202	No	87.6	NA	NA	NA	NA	9.4	PCL-C	Moderate
Wang et al., 2020i	GP	2540<br>2543	No	NA	32.0<br>36.4	36.1<br>42.9	NA	NA	NA	GAD-7, PHQ-9	Mild
Wang et al., 2020j	FHCW	742	Yes	82.5	38.5	21.7	NA	NA	15.4	GAD-7, PHQ-9, IES-R	Overall (ANX) Moderate (DEP & PTSD)
	GHCW	1155			19.8	10.7	NA	NA	6.1		
Wang et al., 2020k	GP	1599	No	66.8	NA	NA	NA	22.9	NA	K-6	Moderate
Wu et al., 2020a	GP	24789	No	46.3	3.6	3.1	NA	NA	NA	HADS	Severe
Wu et al., 2020b	GHCW	304	No	68.1	NA	NA	NA	14.1	NA	K-6	Moderate
Wu et al., 2020c	GP	104	Yes	57.7	NA	NA	NA	NA	9.4	PCL-5	Moderate
		330	No	67.3	NA	NA	NA	NA	7.2		
Xiao et al., 2020	GHCW	958	No	67.2	54.1	57.3	NA	NA	NA	HADS	Mild
Xu et al., 2020	GHCW	8817	No	78.0	0.0	3.2	NA	NA	NA	GAD-7, PHQ-9	Severe
Xing et al., 2020	FHCW	309	No	97.4	2.3	0.6	NA	NA	NA	SAS, SDS	Severe

Xiong et al., 2020	GHCW	231	No	97.3	3.6	1.8	NA	NA	NA	GAD-7, PHQ-9	Severe
Yang et al., 2020a	GHCW	449	No	NA	29.2	NA	NA	NA	NA	SAS	Mild
Yang et al., 2020b	GP	2410	No	49.2	NA	NA	14.9	NA	NA	PSQI	Mild
Yin et al., 2020a	GP	8151	No	57.7	6.7	13.5	NA	NA	NA	GAD-7, PHQ-9	Moderate
Yin et al., 2020b	GHCW	371	No	61.5	NA	NA	NA	NA	3.8	PCL-5	Moderate
Ying et al., 2020	GP	845	No	47.3	33.7	29.4	NA	NA	NA	GAD-7, PHQ-9	Mild
Yu et al., 2020a	GP	1588	No	66.9	NA	NA	NA	22.8	NA	K-6	Moderate
Yu et al., 2020b	GP	1138	No	65.6	NA	NA	29.9	NA	NA	ISI	Moderate
Yu et al., 2020c	FHCW	290	No	64.1	7.3	NA	NA	NA	NA	GAD-7	Severe
Zhan et al., 2020a	FHCW	2667	Yes	97.0	39.8	54.7	NA	NA	NA	GAD-7, PHQ-9	Overall
Zhan et al., 2020b	FHCW	1794	Yes	97.0	NA	NA	52.8	NA	NA	AIS	Overall
Zhang et al., 2020a	GHCW	1563	No	82.5	44.7	50.7	36.1	NA	73.4	GAD-7, PHQ-9, ISI, IES-R	Mild
Zhang et al., 2020b	GP	98	No	65.3	16.3	14.3	NA	NA	NA	GAD-7, PHQ-9	Severe
Zhang et al., 2020c	GP	369	No	45.0	NA	NA	NA	0.0	NA	K-6	Moderate
Zhang et al., 2020d	GHCW	927	No	73.1	13.0	12.2	38.4	NA	NA	GAD-2, PHQ-2, ISI	Mild (INS)<br>Moderate (ANX & DEP)
	GP	1255		57.6	8.5	9.5	30.5	NA	NA		
Zhang et al., 2020e	GP	263	No	59.7	NA	NA	NA	NA	7.6	IES-R	Moderate
Zhang et al., 2020f	FHCW	966	No	76.4	10.7	17.3	NA	NA	NA	GAD-7, PHQ-9	Moderate
Zhang et al., 2020g	FHCW	421	Yes	85.1	14.6	16.2	2.8	NA	22.6	HADS, ISI<br>PCL-C	Moderate (ANX, DEP, & PTSD)<br>Severe (INS)
	GP	221						NA	17.7		
Zhang et al., 2020h	GP	1342	No	62.7	NA	13.60	NA	NA	NA	PHQ-9	Moderate
Zhang et al., 2020i	GP	123768	No	29.4	0.3	0.1	NA	NA	NA	SAS, SDS	Severe
Zhang et al., 2020j	FHCW	269	No	47.1	5.6	4.8	7.8	NA	NA	GAD-7, PHQ-9, ISI	Severe
	GP	2640			4.4	3.1	5.6	NA	NA		
Zhao et al., 2020a	FHCW	972	No	62.7	16.9	10.3	11.2	NA	NA	GAD-7, PHQ-9, ISI	Moderate
Zhao et al., 2020b	GP	1501	No	NA	15.8	14.8	NA	NA	NA	GAD-2, PHQ-2	Moderate
Zhao et al., 2020c	GP	1630	No	NA	0.8	NA	36.4	NA	NA	SAS, PSQI	Mild (INS)<br>Severe (ANX)

Zhou et al., 2020a	FHCW	1931	No	12.0	NA	NA	18.4	NA	NA	PSQI	Moderate
Zhou et al., 2020b	FHCW	606	No	81.2	45.4	57.6	32.0	NA	NA	GAD-7, PHQ-9, ISI	Mild
	GP	1099		69.4	33.8	47.6	25.1	NA	NA		
Zhu et al., 2020a	GP	5281	No	85.0	24.1	13.5	NA	NA	NA	GAD-7, PHQ-9	Overall (ANX)<br>Moderate (DEP)
Zhu et al., 2020b	GHCW	453	No	94.9	28.6	NA	NA	NA	NA	SAS	Moderate
Zhu et al., 2020c	FHCW	165	No	83.0	11.4	45.6	NA	NA	NA	SAS, SDS	Mild
Zhu et al., 2020d	FHCW	320	No	63.7	19.7	21.3	NA	NA	NA	GAD-7, PHQ-9	Mild

Note: ANX= Anxiety, FHCW=Frontline Healthcare Worker, GHCW = General Healthcare Worker, GP = General Population, INS= Insomnia, DEP= Depression, DIS=Distress, NA= Not available, Mild = Above Mild, Moderate = Above Moderate, Severe = Above severe, Overall = Overall

Table S3. **The definition and instruments of mental health symptoms and the results of subgroup analyses on instruments in the systematic review**

Instrument	Frequency	Percent	Prevalence	Citation
**Anxiety:** an emotion characterized by apprehension and somatic symptoms of tension in which an individual anticipates impending danger, catastrophe, or misfortune	123	100		
GAD (GAD-7/GAD-2, Generalized Anxiety Disorder scale - 7-item/2-item)	76	61.8	14%, 95% CI: 12% – 17%	(133, 134)
SAS (Self-rating Anxiety Scale)	29	23.6	2%, 95% CI: 2% – 3%	(135)
HADS (Hospital Anxiety Depression Scale)	7	5.7	21%, 95% CI: 8% – 39%	(136)
DASS-21 (Depression Anxiety Stress Scale)	6	4.9	7%, 95% CI: 1% – 15%	(137)
HAMA (Hamilton Anxiety Rating Scale)	4	3.3	13%, 95% CI: 2% – 32%	(138)
BAI (Beck Anxiety Inventory)	1	0.8		(139)
**Depression:** a fluctuation in normal mood ranging from unhappiness and discontent to an extreme feeling of sadness, pessimism, and despondency	117	100		
PHQ (PHQ-9/PHQ-2, The Patient Health Questionnaire depression scale - 9-item/2-item)	76	65.0	15%, 95% CI: 13% – 17%	(134, 140)
SDS (Self-rating Depression Scale)	17	14.5	4%, 95% CI: 2% – 5%	(141, 142)
DASS-21 (Depression Anxiety Stress Scale)	7	6.0	6%, 95% CI: 1% – 13%	(137)
HADS (Hospital Anxiety Depression Scale)	7	6.9	26%, 95% CI: 9% – 48%	(136)
CES-D (CES-D-20/9/10, The Center for Epidemiologic Studies Depression Scale – 20 items/9 items/10 items)	5	4.2	6%, 95% CI: 1% – 31%	(143)
HAMD (Hamilton Depression Scale)	3	2.6	7%, 95% CI: 1% – 33%	(144)
BDI-II (Beck Depression Inventory - Second Edition)	1	0.8	NA	(145)
WHO-5 (The 5-item World Health Organization Well-Being Index)	1	0.8	NA	(146)
**Distress:** the negative stress response, involving excessive levels of stimulation: a type of stress that results from being overwhelmed by demands, losses, or perceived threats	9	100		
K-6 (the six-item Kessler mental distress scale)	5	50.0	13%, 95% CI: 6% – 23%	(147)

IES-R (Impact of Event Scale - Revised)	1	10.0	NA	(148)
CPDI (COVID-19 Peritraumatic Distress Index)	1	10.0	NA	(31)
GHQ-12 (General Health Questionnaire)	1	10.0	NA	(149)
HEI (Huaxi Emotional-Distress Index)	1	10.0	NA	(150)
**Insomnia:** difficulties in initiating or maintaining a restorative sleep to result in fatigue, the severity or persistence of which causes clinically significant distress or impairment in functioning	57	100		
ISI (Insomnia Severity Index)	35	66.0	12%, 95% CI: 9% – 15%	(151)
PSQI (Pittsburgh Sleep Quality Index)	14	26.4	26%, 95% CI: 19% – 35%	(152, 153)
AIS (Athens Insomnia Scale)	4	7.6	NA	(154)
**PTSD:** a disorder that result when an individual lives through or witnesses an event in which he or she believes that there is a threat to life or physical integrity and safety and experiences fear, terror, or helplessness	31	100		
IES-R (Impact of Event Scale – Revised)	13	41.9	31%, 95% CI: 16% – 48%	(155-157)
PCL-5 (the Posttraumatic Stress Disorder Checklist for DSM-5)	8	25.8	19%, 95% CI: 3% – 44%	(158)
PCL-C (Posttraumatic Stress Disorder Checklist-Civilian Version)	8	25.8	10%, 95% CI: 5% – 16%	(159-161)
IES-6 (Impact of Event Scale – 6 items)	1	3.2	NA	(162)
PC-PTSD (The Primary Care PTSD Screen for DSM-5)	1	3.2	NA	(163)

References

1. Cao J, Wei J, Zhu H, Duan Y, Geng W, Hong X, et al. A Study of Basic Needs and Psychological Wellbeing of Medical Workers in the Fever Clinic of a Tertiary General Hospital in Beijing during the COVID-19 Outbreak. *Psychother Psychosom* (2020) 89(4):252-4. Epub 2020/04/01. doi: 10.1159/000507453. PubMed PMID: 32224612; PubMed Central PMCID: PMCPMC7179543.
2. Dai Y, Hu, G., Xiong, H., Qiu, H., & Yuan, X. Psychological impact of the coronavirus disease 2019 (COVID-19) outbreak on healthcare workers in China. *medRxiv* (2020). doi: 10.1101/2020.03.03.20030874.
3. Du J, Dong L, Wang T, Yuan C, Fu R, Zhang L, et al. Psychological symptoms among frontline healthcare workers during COVID-19 outbreak in Wuhan. *General hospital psychiatry* (2020). Epub 2020/05/10. doi: 10.1016/j.genhosppsych.2020.03.011. PubMed PMID: 32381270; PubMed Central PMCID: PMCPMC7194721.
4. Gao J, Zheng, P., Jia, Y., Chen, H., Mao, Y., Chen, S., . . . Dai, J. Mental health problems and social media exposure during COVID-19 outbreak. *PLoS ONE* (2020) 15(4):e0231924. doi: 10.1371/journal.pone.0231924.
5. Guo J, Liao, L., Wang, B., Li, X., Guo, L., Tong, Z., ... Gu, Y. Psychological effects of COVID-19 on hospital staff: a national cross-sectional survey of China mainland. *SSRN* (2020). doi: 10.2139/ssrn.3550050.
6. Lai J, Ma S, Wang Y, Cai Z, Hu J, Wei N, et al. Factors Associated With Mental Health Outcomes Among Health Care Workers Exposed to Coronavirus Disease 2019. *JAMA Network Open* (2020) 3(3):e203976. Epub 2020/03/24. doi: 10.1001/jamanetworkopen.2020.3976. PubMed PMID: 32202646; PubMed Central PMCID: PMCPMC7090843.
7. Lei L, Huang X, Zhang S, Yang J, Yang L, Xu M. Comparison of Prevalence and Associated Factors of Anxiety and Depression Among People Affected by versus People Unaffected by Quarantine During the COVID-19 Epidemic in Southwestern China. *Med Sci Monit* (2020) 26:e924609. Epub 2020/04/27. doi: 10.12659/MSM.924609. PubMed PMID: 32335579; PubMed Central PMCID: PMCPMC7199435.
8. Li X, Yu, H., Bian, G., Hu, Z., Liu, X., Zhou, Q., . . . Zhou, D. Prevalence, risk factors, and clinical correlates of insomnia in volunteer and at home medical staff during the COVID-19. *Brain Behav Immun* (2020) 87:140-1. doi: 10.1016/j.bbi.2020.05.008.
9. Liu C-Y, Yang, Y.-z., Zhang, X.-M., Xu, X., Dou, Q.-L., Zhang, W.-W., & Cheng, A. S. K. The prevalence and influencing factors in anxiety in medical workers fighting COVID-19 in China: a cross-sectional survey. *Epidemiology and Infection* (2020) 148. doi: 10.1017/s0950268820001107.
10. Liu D, Ren, Y., Yan, F., Li, Y., Xu, X., Yu, X., . . . Tan, S. Psychological Impact and Predisposing Factors of the Coronavirus Disease 2019 (COVID-19) Pandemic on General Public in China. *Lancet Psychiatry* (in press). doi: 10.2139/ssrn.3551415.
11. Liu N, Zhang F, Wei C, Jia Y, Shang Z, Sun L, et al. Prevalence and predictors of PTSS during COVID-19 outbreak in China hardest-hit areas: Gender differences matter. *Psychiatry Res* (2020) 287:112921. Epub 2020/04/03. doi: 10.1016/j.psychres.2020.112921. PubMed PMID: 32240896; PubMed Central PMCID: PMCPMC7102622 of interest.
12. Liu Z, Han B, Jiang RM, Huang YQ, Ma C, Wen J, et al. Mental health status of doctors and nurses during COVID-19 epidemic in China. *Preprint with The Lancet, SSRN* (2020). doi: 10.2139/ssrn.3551329.

13. Lu W, Wang H, Lin Y, Li L. Psychological status of medical workforce during the COVID-19 pandemic: A cross-sectional study. *Psychiatry Res* (2020) 288. doi: 10.1016/j.psychres.2020.112936.
14. Ni MY, Yang L, Leung CMC, Li N, Yao X, Wang Y, et al. Mental health, risk factors, and social media use during the COVID-19 epidemic and cordon sanitaire among the community and health professionals in Wuhan, China: Cross-sectional survey. *JMIR Ment Health* (2020) 7(5):e19009. doi: 10.2196/19009.
15. Qi J, Xu, J., Li, B.-Z., Huang, J.-S., Yang, Y., Zhang, Z.-T., . . . Zhang, X. The evaluation of sleep disturbances for Chinese frontline medical workers under the outbreak of COVID-19. *Sleep Medicine* (2020) 72:1-4. doi: 10.1016/j.sleep.2020.05.023.
16. Qian M, Wu, Q., Wu, P., Hou, Z., Liang, Y., Cowling, B. J., & Yu, H. Psychological responses, behavioral changes and public perceptions during the early phase of the COVID-19 outbreak in China: a population based cross-sectional survey. *medRxiv* (2020). doi: 10.1101/2020.02.18.20024448.
17. Sun L, Sun, Z., Wu, L., Zhu, Z., Zhang, F., Shang, Z., . . . Liu, W. Prevalence and Risk Factors of Acute Posttraumatic Stress Symptoms during the COVID-19 Outbreak in Wuhan, China. *medRxiv* (2020). doi: 10.1101/2020.03.06.20032425.
18. Wang H, Xia Q, Xiong Z, Li Z, Xiang W, Yuan Y, et al. The psychological distress and coping styles in the early stages of the 2019 coronavirus disease (COVID-19) epidemic in the general mainland Chinese population: A web-based survey. *PLoS One* (2020) 15(5):e0233410. Epub 2020/05/15. doi: 10.1371/journal.pone.0233410. PubMed PMID: 32407409; PubMed Central PMCID: PMCPMC7224553.
19. Wang Y, Di Y, Ye J, Wei W. Study on the public psychological states and its related factors during the outbreak of coronavirus disease 2019 (COVID-19) in some regions of China. *Psychol Health Med* (2020):1-10. Epub 2020/04/01. doi: 10.1080/13548506.2020.1746817. PubMed PMID: 32223317.
20. Yu H, Li M, Li Z, Xiang W, Yuan Y, Liu Y, et al. Coping style, social support and psychological distress in the general Chinese population in the early stages of the COVID-2019 epidemic. *SSRN* (2020). doi: 10.2139/ssrn.3556633.
21. Zhang C, Yang L, Liu S, Ma S, Wang Y, Cai Z, et al. Survey of Insomnia and Related Social Psychological Factors Among Medical Staff Involved in the 2019 Novel Coronavirus Disease Outbreak. *Frontiers in Psychiatry* (2020) 11:306. Epub 2020/04/30. doi: 10.3389/fpsyt.2020.00306. PubMed PMID: 32346373; PubMed Central PMCID: PMCPMC7171048.
22. Zhang J, Lu, H., Zeng, H., Zhang, S., Du, Q., Jiang, T., & Dua, B. The differential psychological distress of populations affected by the COVID-19 pandemic. *Brain Behav Immun* (2020) 87:49–50. doi: 10.1016/j.bbi.2020.04.031.
23. Zhang SX, Liu J, Afshar Jahanshahi A, Nawaser K, Yousefi A, Li J, et al. At the height of the storm: Healthcare staff's health conditions and job satisfaction and their associated predictors during the epidemic peak of COVID-19. *Brain Behav Immun* (2020) 87:144-6. Epub 2020/05/11. doi: 10.1016/j.bbi.2020.05.010. PubMed PMID: 32387345; PubMed Central PMCID: PMCPMC7199703.
24. Zhang WR, Wang K, Yin L, Zhao WF, Xue Q, Peng M, et al. Mental Health and Psychosocial Problems of Medical Health Workers during the COVID-19 Epidemic in China. *Psychother Psychosom* (2020) 89(4):242-50. Epub 2020/04/10. doi: 10.1159/000507639. PubMed PMID: 32272480; PubMed Central PMCID: PMCPMC7206349.
25. Zhang Y, Ma ZF. Impact of the COVID-19 Pandemic on Mental Health and Quality of Life among Local Residents in Liaoning Province, China: A Cross-Sectional Study. *Int J*

Environ Res Public Health (2020) 17(7). Epub 2020/04/05. doi: 10.3390/ijerph17072381. PubMed PMID: 32244498; PubMed Central PMCID: PMCPMC7177660.
26. Zhu Z, Xu, S., Wang, H., Liu, Z., Wu, J., Li, G., . . . Wang, W. COVID-19 in Wuhan: Immediate Psychological Impact on 5062 Health Workers. *medRxiv* (2020). doi: 10.1101/2020.02.20.20025338.
27. Tan W, Hao F, McIntyre RS, Jiang L, Jiang X, Zhang L, et al. Is returning to work during the COVID-19 pandemic stressful? A study on immediate mental health status and psychoneuroimmunity prevention measures of Chinese workforce. *Brain Behav Immun* (2020) 87:84-92. Epub 2020/04/27. doi: 10.1016/j.bbi.2020.04.055. PubMed PMID: 32335200; PubMed Central PMCID: PMCPMC7179503.
28. Chen Y, Zhou H, Zhou Y, Zhou F. Prevalence of self-reported depression and anxiety among pediatric medical staff members during the COVID-19 outbreak in Guiyang, China. *Psychiatry Res* (2020) 288:113005. Epub 2020/04/22. doi: 10.1016/j.psychres.2020.113005. PubMed PMID: 32315886; PubMed Central PMCID: PMCPMC7160637 interest.
29. Li G, Miao, J, Wang, H, Xu, S, Sun, W, Fan, Y, et al. Psychological impact on women health workers involved in COVID-19 outbreak in Wuhan: a cross-sectional study. *Journal of Neurology, Neurosurgery & Psychiatry* (2020). doi: 10.1136/jnnp-2020-323134.
30. Li Y, Qin Q, Sun Q, Sanford LD, Vgontzas AN, Tang X. Insomnia and psychological reactions during the COVID-19 outbreak in China. *J Clin Sleep Med* (2020) 16(8):1417-8. Epub 2020/05/01. doi: 10.5664/jcsm.8524. PubMed PMID: 32351206; PubMed Central PMCID: PMCPMC7446072.
31. Qiu J, Shen B, Zhao M, Wang Z, Xie B, Xu Y. A nationwide survey of psychological distress among Chinese people in the COVID-19 epidemic: implications and policy recommendations. *Gen Psychiatry* (2020) 33(2):e100213. Epub 2020/03/28. doi: 10.1136/gpsych-2020-100213. PubMed PMID: 32215365; PubMed Central PMCID: PMCPMC7061893.
32. Wang S, Xie L, Xu Y, Yu S, Yao B, Xiang D. Sleep disturbances among medical workers during the outbreak of COVID-2019. *Occup Med (Lond)* (2020) 70(5):364-9. Epub 2020/05/07. doi: 10.1093/occmed/kqaa074. PubMed PMID: 32372077; PubMed Central PMCID: PMCPMC7239094.
33. Zhu J, Sun L, Zhang L, Wang H, Fan A, Yang B, et al. Prevalence and Influencing Factors of Anxiety and Depression Symptoms in the First-Line Medical Staff Fighting Against COVID-19 in Gansu. *Frontiers in Psychiatry* (2020) 11:386. Epub 2020/05/16. doi: 10.3389/fpsyt.2020.00386. PubMed PMID: 32411034; PubMed Central PMCID: PMCPMC7202136.
34. Zhu S, Wu Y, Zhu CY, Hong WC, Yu ZX, Chen ZK, et al. The immediate mental health impacts of the COVID-19 pandemic among people with or without quarantine managements. *Brain Behav Immun* (2020) 87:56-8. Epub 2020/04/22. doi: 10.1016/j.bbi.2020.04.045. PubMed PMID: 32315758; PubMed Central PMCID: PMCPMC7165285 competing financial interests or personal relationships that could have appeared to influence the work reported in this paper.
35. Zhou Y, Yang Y, Shi T, Song Y, Zhou Y, Zhang Z, et al. Prevalence and Demographic Correlates of Poor Sleep Quality Among Frontline Health Professionals in Liaoning Province, China During the COVID-19 Outbreak. *Frontiers in Psychiatry* (2020) 11:520. Epub 2020/07/01. doi: 10.3389/fpsyt.2020.00520. PubMed PMID: 32595534; PubMed Central PMCID: PMCPMC7304227.
36. Que J, Shi L, Deng J, Liu J, Zhang L, Wu S, et al. Psychological impact of the COVID-19 pandemic on healthcare workers: a cross-sectional study in China. *Gen*

Psychiatry (2020) 33(3):e100259. Epub 2020/07/01. doi: 10.1136/gpsych-2020-100259. PubMed PMID: 32596640; PubMed Central PMCID: PMCPMC7299004.
37. Tu ZH, He JW, Zhou N. Sleep quality and mood symptoms in conscripted frontline nurse in Wuhan, China during COVID-19 outbreak: A cross-sectional study. *Medicine (Baltimore)* (2020) 99(26):e20769. Epub 2020/06/27. doi: 10.1097/MD.0000000000020769. PubMed PMID: 32590755; PubMed Central PMCID: PMCPMC7328950.
38. An Y, Yang Y, Wang A, Li Y, Zhang Q, Cheung T, et al. Prevalence of depression and its impact on quality of life among frontline nurses in emergency departments during the COVID-19 outbreak. *J Affect Disord* (2020) 276:312-5. Epub 2020/09/03. doi: 10.1016/j.jad.2020.06.047. PubMed PMID: 32871661; PubMed Central PMCID: PMCPMC7361044.
39. Wang H, Huang D, Huang H, Zhang J, Guo L, Liu Y, et al. The psychological impact of COVID-19 pandemic on medical staff in Guangdong, China: a cross-sectional study. *Psychol Med* (2020):1-9. Epub 2020/07/07. doi: 10.1017/S0033291720002561. PubMed PMID: 32624037; PubMed Central PMCID: PMCPMC7371926.
40. Song X, Fu, W., Liu, X., Luo, Z., Wang, R., Zhou, N., . . . Lv, C. . Mental health status of medical staff in emergency departments during the Coronavirus disease 2019 epidemic in China. *Brain Behav Immun* (2020) 88:60-5. Epub 2020/06/09. doi: 10.1016/j.bbi.2020.06.002. PubMed PMID: 32512134; PubMed Central PMCID: PMCPMC7273140.
41. Han L, Wong FKY, She DLM, Li SY, Yang YF, Jiang MY, et al. Anxiety and Depression of Nurses in a North West Province in China During the Period of Novel Coronavirus Pneumonia Outbreak. *J Nurs Scholarsh* (2020). Epub 2020/07/12. doi: 10.1111/jnu.12590. PubMed PMID: 32652884; PubMed Central PMCID: PMCPMC7405411.
42. Yin X, Wang J, Feng J, Chen Z, Jiang N, Wu J, et al. The Impact of the Corona Virus Disease 2019 Outbreak on Chinese Residents' Mental Health. *SSRN* (2020). doi: 10.2139/ssrn.3556680.
43. Dong Z-Q, Ma J, Hao Y-N, Shen X-L, Liu F, Gao Y, et al. The social psychological impact of the COVID-19 pandemic on medical staff in China: A cross-sectional study. *European Psychiatry* (2020) 63(1). doi: 10.1192/j.eurpsy.2020.59.
44. Elhai JD, Yang H, McKay D, Asmundson GJ. COVID-19 anxiety symptoms associated with problematic smartphone use severity in Chinese adults. *J Affect Disord* (2020). doi: 10.1016/j.jad.2020.05.080.
45. Fang X, Zhang J, Teng C, Zhao K, Su K-P, Wang Z, et al. Depressive symptoms in the front-line non-medical workers during the COVID-19 outbreak in Wuhan. *J Affect Disord* (2020) 276:441-5. doi: 10.1016/j.jad.2020.06.078.
46. Feng Z, Xu L, Cheng P, Zhang L, Li L-J, Li W-H. The psychological impact of COVID-19 on the families of first-line rescuers. *Indian journal of psychiatry* (2020) 62(Suppl 3):S438. doi: 10.4103/psychiatry.IndianJPsychiatry_1057_20.
47. Fong BY, Wong M, Law VT, Lo MF, Ng TK, Yee HH, et al. Relationships between Physical and Social Behavioural Changes and the Mental Status of Homebound Residents in Hong Kong during the COVID-19 Pandemic. *Int J Environ Res Public Health* (2020) 17(18):6653. doi: 10.3390/ijerph17186653.
48. Fu W, Wang C, Zou L, Guo Y, Lu Z, Yan S, et al. Psychological health, sleep quality, and coping styles to stress facing the COVID-19 in Wuhan, China. *Translational psychiatry* (2020) 10(1):1-9. doi: 10.1038/s41398-020-00913-3.
49. Guo J, Feng XL, Wang XH, van IJzendoorn MH. Coping with COVID-19: Exposure to COVID-19 and Negative Impact on Livelihood Predict Elevated Mental Health Problems

in Chinese Adults. *Int J Environ Res Public Health* (2020) 17(11):3857. doi: 10.3390/ijerph17113857.
50. Hong S, Ai M, Xu X, Wang W, Chen J, Zhang Q, et al. Immediate psychological impact on nurses working at 42 government-designated hospitals during COVID-19 outbreak in China: A cross-sectional study. *Nursing outlook* (2020). doi: 10.1016/j.outlook.2020.07.007.
51. Hou F, Bi F, Jiao R, Luo D, Song K. Gender differences of depression and anxiety among social media users during the COVID-19 outbreak in China: a cross-sectional study. *BMC public health* (2020) 20(1):1-11. doi: 10.1186/s12889-020-09738-7.
52. Hu D, Kong Y, Li W, Han Q, Zhang X, Zhu LX, et al. Frontline Nurses' Burnout, Anxiety, Depression, and Fear Statuses and Their Associated Factors During the COVID-19 Outbreak in Wuhan, China: A Big-Scale Cross-Sectional Study. *eClinical Medicine* (2020). doi: 10.1016/j.eclinm.2020.100424.
53. Hu N, Li Y, He S-S, Wang L-L, Wei Y-Y, Yin L, et al. Impact of the Family Environment on the Emotional State of Medical Staff During the COVID-19 Outbreak: The Mediating Effect of Self-Efficacy. *Frontiers in Psychology* (2020) 11. doi: 10.3389/fpsyg.2020.576515.
54. Huang J, Liu F, Teng Z, Chen J, Zhao J, Wang X, et al., editors. Public behavior change, perceptions, depression, and anxiety in relation to the COVID-19 outbreak. *Open Forum Infectious Diseases*; 2020: Oxford University Press US.
55. Huang L, Wang Y, Liu J, Ye P, Chen X, Xu H, et al. Factors influencing anxiety of health care workers in the radiology department with high exposure risk to COVID-19. *Medical Science Monitor: International Medical Journal of Experimental and Clinical Research* (2020) 26:e926008-1. doi: 10.12659/MSM.926008.
56. Huang Y, Wang Y, Zeng L, Yang J, Song X, Rao W, et al. Prevalence and Correlation of Anxiety, Insomnia and Somatic Symptoms in a Chinese Population During the COVID-19 Epidemic. *Frontiers in Psychiatry* (2020) 11:894. doi: 10.3389/fpsyt.2020.568329.
57. Jin Z, Zhao K-b, Xia Y-y, Chen R-j, Yu H, Tamutana TT, et al. Relationship Between Psychological Responses and the Appraisal of Risk Communication During the Early Phase of the COVID-19 Pandemic: A Two-Wave Study of Community Residents in China. *Frontiers in Public Health* (2020) 8. doi: 10.3389/fpubh.2020.550220.
58. Juan Y, Yuanyuan C, Qiuxiang Y, Cong L, Xiaofeng L, Yundong Z, et al. Psychological distress surveillance and related impact analysis of hospital staff during the COVID-19 epidemic in Chongqing, China. *Comprehensive Psychiatry* (2020) 103:152198. doi: 10.1016/j.comppsych.2020.152198.
59. Lam SC, Arora T, Grey I, Suen LKP, Huang EY-z, Li D, et al. Perceived risk and protection from infection and depressive symptoms among healthcare workers in mainland China and Hong Kong during COVID-19. *Frontiers in Psychiatry* (2020) 11:686. doi: 10.3389/fpsyt.2020.00686.
60. Leng M, Wei L, Shi X, Cao G, Wei Y, Xu H, et al. Mental distress and influencing factors in nurses caring for patients with COVID-19. *Nursing in Critical Care* (2020). doi: 10.1111/nicc.12528.
61. Li Q. Psychosocial and coping responses toward 2019 coronavirus diseases (COVID-19): a cross-sectional study within the Chinese general population. *QJM: An International Journal of Medicine* (2020) 113(10):731-8. doi: 10.1093/qjmed/hcaa226.
62. Li Q, Chen J, Xu G, Zhao J, Yu X, Wang S, et al. The Psychological Health Status of Healthcare Workers During the COVID-19 Outbreak: A Cross-Sectional Survey Study in

Guangdong, China. *Frontiers in Public Health* (2020) 8:572. doi: 10.3389/fpubh.2020.562885.
63. Li Q, Miao Y, Zeng X, Tarimo CS, Wu C, Wu J. Prevalence and factors for anxiety during the coronavirus disease 2019 (COVID-19) epidemic among the teachers in China. *J Affect Disord* (2020) 277:153-8. doi: 10.1016/j.jad.2020.08.017.
64. Li R, Chen Y, Lv J, Liu L, Zong S, Li H, et al. Anxiety and related factors in frontline clinical nurses fighting COVID-19 in Wuhan. *Medicine* (2020) 99(30). doi: 10.1097/MD.0000000000021413.
65. Li X, Li S, Xiang M, Fang Y, Qian K, Xu J, et al. The prevalence and risk factors of PTSD symptoms among medical assistance workers during the COVID-19 pandemic. *Journal of Psychosomatic Research* (2020) 139:110270. doi: 10.1016/j.jpsychores.2020.110270.
66. Li X, Zhou Y, Xu X. Factors associated with the psychological well-being among front-line nurses exposed to COVID-2019 in China: A predictive study. *Journal of nursing management* (2020). doi: 10.1111/jonm.13146.
67. Liang Y, Wu K, Zhou Y, Huang X, Zhou Y, Liu Z. Mental health in frontline medical workers during the 2019 novel coronavirus disease epidemic in China: a comparison with the general population. *Int J Environ Res Public Health* (2020) 17(18):6550. doi: 10.3390/ijerph17186550.
68. Lin K, Yang BX, Luo D, Liu Q, Ma S, Huang R, et al. The mental health effects of COVID-19 on health care providers in China. *American Journal of Psychiatry* (2020) 177(7):635-6. doi: 10.1176/appi.ajp.2020.20040374.
69. Lin L-y, Wang J, Ou-yang X-y, Miao Q, Chen R, Liang F-x, et al. The immediate impact of the 2019 novel coronavirus (COVID-19) outbreak on subjective sleep status. *Sleep Medicine* (2020). doi: 10.1016/j.sleep.2020.05.018.
70. Liu M, Zhang H, Huang H. Media exposure to COVID-19 information, risk perception, social and geographical proximity, and self-rated anxiety in China. *BMC Public Health* (2020) 20(1):1-8. doi: 10.1186/s12889-020-09761-8.
71. Liu X, Luo W-T, Li Y, Li C-N, Hong Z-S, Chen H-L, et al. Psychological status and behavior changes of the public during the COVID-19 epidemic in China. *Infectious Diseases of Poverty* (2020) 9(1):1-11. doi: 10.1186/s40249-020-00678-3.
72. Liu Y, Chen H, Zhang N, Wang X, Fan Q, Zhang Y, et al. Anxiety and depression symptoms of medical staff under COVID-19 epidemic in China. *J Affect Disord* (2020) 278:144-8. doi: 10.1016/j.jad.2020.09.004.
73. Liu Y, Wang L, Chen L, Zhang X, Bao L, Shi Y. Mental health status of paediatric medical workers in China during the COVID-19 outbreak. *Frontiers in Psychiatry* (2020) 11. doi: 10.3389/fpsyt.2020.00702.
74. Lu H, Nie P, Qian L. Do quarantine experiences and attitudes towards COVID-19 affect the distribution of mental health in China? A quantile regression analysis. *Applied Research in Quality of Life* (2020):1-18. doi: 10.1007/s11482-020-09851-0.
75. Mi T, Yang X, Sun S, Li X, Tam CC, Zhou Y, et al. Mental Health Problems of HIV Healthcare Providers During the COVID-19 Pandemic: The Interactive Effects of Stressors and Coping. *AIDS and Behavior* (2020):1-10. doi: 10.1007/s10461-020-03073-z.
76. Ni Z, Lebowitz ER, Zou Z, Wang H, Liu H, Shrestha R, et al. Response to the COVID-19 Outbreak in Urban Settings in China. *Journal of Urban Health* (2020):1-12. doi: 10.1007/s11524-020-00498-8.
77. Ning X, Yu F, Huang Q, Li X, Luo Y, Huang Q, et al. The mental health of neurological doctors and nurses in Hunan Province, China during the initial stages of the COVID-19 outbreak. *BMC psychiatry* (2020) 20(1):1-9. doi: 10.1186/s12888-020-02838-z.

78. Pan W, Hu J, Yi L. Mental state of central sterile supply department staff during COVID-19 epidemic and CART analysis. *BMC health services research* (2020) 20(1):1-9. doi: 10.1186/s12913-020-05864-5.
79. Pan X, Xiao Y, Ren D, Xu ZM, Zhang Q, Yang LY, et al. Prevalence of mental health problems and associated risk factors among military healthcare workers in specialized COVID-19 hospitals in Wuhan, China: A cross-sectional survey. *Asia-Pacific Psychiatry* (2020):e12427. doi: 10.1111/appy.12427.
80. Pan Y, Fang Y, Xin M, Dong W, Zhou L, Hou Q, et al. Self-reported compliance with personal preventive measures among Chinese factory workers at the beginning of work resumption following the COVID-19 outbreak: Cross-sectional survey study. *Journal of medical Internet research* (2020) 22(9):e22457. doi: 10.2196/22457.
81. Qian M, Wu Q, Wu P, Hou Z, Liang Y, Cowling BJ, et al. Anxiety levels, precautionary behaviours and public perceptions during the early phase of the COVID-19 outbreak in China: a population-based cross-sectional survey. *BMJ open* (2020) 10(10):e040910. doi: 10.1136/bmjopen-2020-040910.
82. Ren Z, Zhou Y, Liu Y. The psychological burden experienced by Chinese citizens during the COVID-19 outbreak: prevalence and determinants. *BMC Public Health* (2020) 20(1):1-10. doi: 10.1186/s12889-020-09723-0.
83. Shi L, Lu Z-A, Que J-Y, Huang X-L, Liu L, Ran M-S, et al. Prevalence of and risk factors associated with mental health symptoms among the general population in China during the coronavirus disease 2019 pandemic. *JAMA network open* (2020) 3(7):e2014053-e. doi: 10.1001/jamanetworkopen.2020.14053.
84. Si M, Su X, Jiang Y, Wang W, Gu X-f, Ma L, et al. The psychological impact of COVID-19 on medical care workers in China. *SSRN* (2020). doi: 10.2139/ssrn.3592642.
85. Song L, Wang Y, Li Z, Yang Y, Li H. Mental health and work attitudes among people resuming work during the Covid-19 pandemic: A cross-sectional study in China. *Int J Environ Res Public Health* (2020) 17(14):5059. doi: 10.3390/ijerph17145059.
86. Su J, Chen X, Yang N, Sun M, Zhou L. Proximity to people with COVID-19 and anxiety among community residents during the epidemic in Guangzhou, China. *BJPsych open* (2020) 6(4). doi: 10.1192/bjo.2020.59.
87. Sun H, Wang S, Wang W, Han G, Liu Z, Wu Q, et al. Correlation between emotional intelligence and negative emotions of front-line nurses during the COVID-19 epidemic: a cross-sectional study. *Journal of clinical nursing* (2020). doi: 10.1111/jocn.15548.
88. Sun Q, Lu N. Social Capital and Mental Health among Older Adults Living in Urban China in the Context of COVID-19 Pandemic. *Int J Environ Res Public Health* (2020) 17(21):7947. doi: 10.3390/ijerph17217947.
89. Teng Z, Wei Z, Qiu Y, Tan Y, Chen J, Tang H, et al. Psychological status and fatigue of frontline staff two months after the COVID-19 pandemic outbreak in China: A cross-sectional study. *J Affect Disord* (2020). doi: 10.1016/j.jad.2020.06.032.
90. Wang J, Gong Y, Chen Z, Wu J, Feng J, Yan S, et al. Sleep disturbances among Chinese residents during the Coronavirus Disease 2019 outbreak and associated factors. *Sleep medicine* (2020) 74:199-203. doi: 10.1016/j.sleep.2020.08.002.
91. Wang L-Q, Zhang M, Liu G-M, Nan S-Y, Li T, Xu L, et al. Psychological impact of coronavirus disease (2019)(COVID-19) epidemic on medical staff in different posts in China: A multicenter study. *J Psychiatr Res* (2020) 129:198-205. doi: 10.1016/j.jpsychires.2020.07.008.
92. Wang S, Zhang Y, Ding W, Meng Y, Hu H, Liu Z, et al. Psychological distress and sleep problems when people are under interpersonal isolation during an epidemic: a

nationwide multicenter cross-sectional study. *European Psychiatry* (2020) 63(1). doi: 10.1192/j.eurpsy.2020.78.
93. Wang W, Song W, Xia Z, He Y, Tang L, Hou J, et al. Sleep disturbance and psychological profiles of medical staff and non-medical staff during the early outbreak of COVID-19 in Hubei Province, China. *Frontiers in Psychiatry* (2020) 11:733. doi: 10.3389/fpsyt.2020.00733.
94. Wang Y-X, Guo H-T, Du X-W, Song W, Lu C, Hao W-N. Factors associated with post-traumatic stress disorder of nurses exposed to corona virus disease 2019 in China. *Medicine* (2020) 99(26). doi: 10.1097/MD.0000000000020965.
95. Wang Y, Hu Z, Feng Y, Wilson A, Chen R. Changes in network centrality of psychopathology symptoms between the COVID-19 outbreak and after peak. *Molecular psychiatry* (2020) 25(12):3140-9. doi: 10.1038/s41380-020-00881-6.
96. Wang Y, Ma S, Yang C, Cai Z, Hu S, Zhang B, et al. Acute psychological effects of Coronavirus Disease 2019 outbreak among healthcare workers in China: a cross-sectional study. *Translational psychiatry* (2020) 10(1):1-10. doi: 10.1038/s41398-020-01031-w.
97. Wu M, Han H, Lin T, Chen M, Wu J, Du X, et al. Prevalence and risk factors of mental distress in China during the outbreak of COVID-19: A national cross-sectional survey. *Brain and behavior* (2020) 10(11):e01818. doi: 10.1002/brb3.1818.
98. Wu S, Li Z, Li Z, Xiang W, Yuan Y, Liu Y, et al. The mental state and risk factors of Chinese medical staff and medical students in early stages of the COVID-19 epidemic. *Comprehensive psychiatry* (2020) 102:152202. doi: 10.1016/j.comppsych.2020.152202.
99. Xiao X, Zhu X, Fu S, Hu Y, Li X, Xiao J. Psychological impact of healthcare workers in China during COVID-19 pneumonia epidemic: a multi-center cross-sectional survey investigation. *J Affect Disord* (2020). doi: 10.1016/j.jad.2020.05.081.
100. Xiaoming X, Ming A, Su H, Wo W, Jianmei C, Qi Z, et al. The psychological status of 8817 hospital workers during COVID-19 Epidemic: A cross-sectional study in Chongqing. *J Affect Disord* (2020) 276:555-61. doi: 10.1016/j.jad.2020.07.092.
101. Xing L-q, Xu M-l, Sun J, Wang Q-X, Ge D-d, Jiang M-m, et al. Anxiety and depression in frontline health care workers during the outbreak of Covid-19. *International Journal of Social Psychiatry* (2020):0020764020968119. doi: 10.1177/0020764020968119.
102. Xiong H, Yi S, Lin Y. The psychological status and self-efficacy of nurses during COVID-19 outbreak: a cross-sectional survey. *INQUIRY: The Journal of Health Care Organization, Provision, and Financing* (2020) 57:0046958020957114. doi: 10.1177/0046958020957114.
103. Yang X, Zhang Y, Li S, Chen X. Risk factors for anxiety of otolaryngology healthcare workers in Hubei province fighting coronavirus disease 2019 (COVID-19). *Social psychiatry and psychiatric epidemiology* (2020):1-7. doi: 10.1007/s00127-020-01928-3.
104. Yang Y, Zhu J-f, Yang S-y, Lin H-j, Chen Y, Zhao Q, et al. Prevalence and associated factors of poor sleep quality among Chinese returning workers during the COVID-19 pandemic. *Sleep medicine* (2020) 73:47-52. doi: 10.1016/j.sleep.2020.06.034.
105. Yin Q, Sun Z, Liu T, Ni X, Deng X, Jia Y, et al. Posttraumatic Stress Symptoms of Health Care Workers during the Corona Virus Disease 2019 (COVID-19). *Clinical Psychology & Psychotherapy* (2020). doi: 10.1002/cpp.2477.
106. Ying Y, Ruan L, Kong F, Zhu B, Ji Y, Lou Z. Mental health status among family members of health care workers in Ningbo, China, during the coronavirus disease 2019 (COVID-19) outbreak: a cross-sectional study. *BMC psychiatry* (2020) 20(1):1-10. doi: 10.1186/s12888-020-02784-w.

107. Yu BY-M, Yeung W-F, Lam JC-S, Yuen SC-S, Lam SC, Chung VC-H, et al. Prevalence of sleep disturbances during covid-19 outbreak in an urban Chinese population: a cross-sectional study. *Sleep medicine* (2020) 74:18-24. doi: 10.1016/j.sleep.2020.07.009.
108. Xu H, Li Y, Zeng M, Zhao X, Li T. Psychological Behavior of Frontline Medical Staff in the Use of Preventive Medication for COVID-19: A Cross-Sectional Study. *Frontiers in Psychology* (2020) 11:2104. doi: 10.3389/fpsyg.2020.02104.
109. Zhan Y-x, Zhao S-y, Yuan J, Liu H, Liu Y-f, Gui L-l, et al. Prevalence and Influencing Factors on Fatigue of First-line Nurses Combating with COVID-19 in China: A Descriptive Cross-Sectional Study. *Current medical science* (2020) 40(4):625-35. doi: 10.1007/s11596-020-2226-9.
110. Zhan Y, Liu Y, Liu H, Li M, Shen Y, Gui L, et al. Factors associated with insomnia among Chinese front-line nurses fighting against COVID-19 in Wuhan: A cross-sectional survey. *Journal of nursing management* (2020) 28(7):1525-35. doi: 10.1111/jonm.13094.
111. Zhang C, Peng D, Lv L, Zhuo K, Yu K, Shen T, et al. Individual Perceived Stress Mediates Psychological Distress in Medical Workers During COVID-19 Epidemic Outbreak in Wuhan. *Neuropsychiatric disease and treatment* (2020) 16:2529. doi: 10.2147/NDT.S266151.
112. Zhang H, Shi Y, Jing P, Zhan P, Fang Y, Wang F. Posttraumatic stress disorder symptoms in healthcare workers after the peak of the COVID-19 outbreak: A survey of a large tertiary care hospital in Wuhan. *Psychiatry Res* (2020) 294:113541. doi: 10.1016/j.psychres.2020.113541.
113. Zhang W, Yang X, Zhao J, Yang F, Jia Y, Cui C, et al. Depression and Psychological-Behavioral Responses Among the General Public in China During the Early Stages of the COVID-19 Pandemic: Survey Study. *Journal of medical Internet research* (2020) 22(9):e22227. doi: 10.2196/22227.
114. Zhang X-R, Huang Q-M, Wang X-M, Cheng X, Li Z-H, Wang Z-H, et al. Prevalence of anxiety and depression symptoms, and association with epidemic-related factors during the epidemic period of COVID-19 among 123,768 workers in China: A large cross-sectional study. *J Affect Disord* (2020) 277:495-502. doi: 10.1016/j.jad.2020.08.041.
115. Zhang Y, Wang S, Ding W, Meng Y, Hu H, Liu Z, et al. Status and influential factors of anxiety depression and insomnia symptoms in the work resumption period of COVID-19 epidemic: A multicenter cross-sectional study. *Journal of Psychosomatic Research* (2020) 138:110253. doi: 10.1016/j.jpsychores.2020.110253.
116. Zhao K, Zhang G, Feng R, Wang W, Xu D, Liu Y, et al. Anxiety, depression and insomnia: A cross-sectional study of frontline staff fighting against COVID-19 in Wenzhou, China. *Psychiatry Res* (2020) 292:113304. doi: 10.1016/j.psychres.2020.113304.
117. Zhao SZ, Wong JYH, Luk TT, Wai AKC, Lam TH, Wang MP. Mental health crisis under COVID-19 pandemic in Hong Kong, China. *International Journal of Infectious Diseases* (2020) 100:431-3. doi: 10.1016/j.ijid.2020.09.030.
118. Zhao X, Lan M, Li H, Yang J. Perceived Stress and Sleep Quality Among the Non-diseased General Public in China During the 2019 Coronavirus Disease: A Moderated Mediation Model. *Sleep Medicine* (2020). doi: 10.1016/j.sleep.2020.05.021.
119. Zhou Y, Wang W, Sun Y, Qian W, Liu Z, Wang R, et al. The prevalence and risk factors of psychological disturbances of frontline medical staff in china under the COVID-19 epidemic: Workload should be concerned. *J Affect Disord* (2020) 277:510-4. doi: 10.1016/j.jad.2020.08.059.
120. Zhu W, Wei Y, Meng X, Li J. The mediation effects of coping style on the relationship between social support and anxiety in Chinese medical staff during COVID-19. *BMC health services research* (2020) 20(1):1-7. doi: 10.1186/s12913-020-05871-6.

121. Wu L, Guo X, Shang Z, Sun Z, Jia Y, Sun L, et al. China experience from COVID-19: Mental health in mandatory quarantine zones urgently requires intervention. *Brain and Behavior* (2020). doi: 10.1037/tra0000609.
122. Sun Y, Song H, Liu H, Mao F, Sun X, Cao F. Occupational stress, mental health, and self-efficacy among community mental health workers: A cross-sectional study during COVID-19 pandemic. *International Journal of Social Psychiatry* (2020):0020764020972131. doi: 10.1177/0020764020972131.
123. Li L, Sun N, Fei S, Yu L, Chen S, Yang S, et al. Current status of and factors influencing anxiety and depression in front-line medical staff supporting Wuhan in containing the novel coronavirus pneumonia epidemic. *Japan Journal of Nursing Science* (2020):e12398. doi: 10.1111/jjns.12398.
124. Lu P, Li X, Lu L, Zhang Y. The psychological states of people after Wuhan eased the lockdown. *PLoS ONE* (2020) 15(11):e0241173. doi: 10.1371/journal.pone.0241173.
125. Ben-Ezra M, Sun S, Hou WK, Goodwin R. The association of being in quarantine and related COVID-19 recommended and non-recommended behaviors with psychological distress in Chinese population. *J Affect Disord* (2020) 275:66-8. doi: 10.1016/j.jad.2020.06.026. PubMed PMID: 32658825.
126. Chen H, Zhao X, Zeng M, Li J, Ren X, Zhang M, et al. Collective self-esteem and perceived stress among the non-infected general public in China during the 2019 coronavirus pandemic: A multiple mediation model. *Pers Individ Dif* (2020) 168:110308. doi: 10.1016/j.paid.2020.110308. PubMed PMID: 32834290.
127. Cai Z, Cui Q, Liu Z, Li J, Gong X, Liu J, et al. Nurses endured high risks of psychological problems under the epidemic of COVID-19 in a longitudinal study in Wuhan China. *J Psychiatr Res* (2020) 131:132-7. doi: 10.1016/j.jpsychires.2020.09.007. PubMed PMID: 32971356.
128. Chen B, Li QX, Zhang H, Zhu JY, Yang X, Wu YH, et al. The psychological impact of COVID-19 outbreak on medical staff and the general public. *Curr Psychol* (2020):1-9. doi: 10.1007/s12144-020-01109-0. PubMed PMID: 33046955.
129. Chen H, Wang B, Cheng Y, Muhammad B, Li S, Miao Z, et al. Prevalence of posttraumatic stress symptoms in health care workers after exposure to patients with COVID-19. *Neurobiol Stress* (2020) 13:100261. doi: 10.1016/j.ynstr.2020.100261. PubMed PMID: 33163588.
130. Chen J, Liu X, Wang D, Jin Y, He M, Ma Y, et al. Risk factors for depression and anxiety in healthcare workers deployed during the COVID-19 outbreak in China. *Soc Psychiatry Psychiatr Epidemiol* (2020) 10:10. doi: 10.1007/s00127-020-01954-1. PubMed PMID: 32914298.
131. Cheng FF, Zhan SH, Xie AW, Cai SZ, Hui L, Kong XX, et al. Anxiety in Chinese pediatric medical staff during the outbreak of Coronavirus Disease 2019: a cross-sectional study. *Transl Pediatr* (2020) 9(3):231-6. doi: 10.21037/tp.2020.04.02. PubMed PMID: 32775241.
132. Choi EPH, Hui BPH, Wan EYF. Depression and Anxiety in Hong Kong during COVID-19. *Int J Environ Res Public Health* (2020) 17(10):25. doi: 10.3390/ijerph17103740. PubMed PMID: 32466251.
133. Spitzer RL, Kroenke K, Williams JB, Löwe B. A brief measure for assessing generalized anxiety disorder: the GAD-7. *Archives of internal medicine* (2006) 166(10):1092-7. doi: 10.1001/archinte.166.10.1092.
134. Kroenke K, Spitzer RL, Williams JB, Löwe B. An ultra-brief screening scale for anxiety and depression: the PHQ–4. *Psychosomatics* (2009) 50(6):613-21. doi: 10.1016/S0033-3182(09)70864-3.

135. Zung WW. A rating instrument for anxiety disorders. *Psychosomatics: Journal of Consultation and Liaison Psychiatry* (1971). doi: 10.1016/S0033-3182(71)71479-0.
136. Zigmond AS, Snaith RP. The hospital anxiety and depression scale. *Acta psychiatrica scandinavica* (1983) 67(6):361-70. doi: 10.1111/j.1600-0447.1983.tb09716.x.
137. Antony MM, Bieling PJ, Cox BJ, Enns MW, Swinson R. Psychometric properties of the 42-item and 21-item versions of the Depression Anxiety Stress Scales in clinical groups and a community sample. *Psychological assessment* (1998) 10(2):176. doi: 10.1037/1040-3590.10.2.176.
138. Matza LS, Morlock R, Sexton C, Malley K, Feltner D. Identifying HAM-A cutoffs for mild, moderate, and severe generalized anxiety disorder. *International Journal of Methods in Psychiatric Research* (2010) 19(4):223-32. doi: 10.1002/mpr.323.
139. Cheng SK-W, Wong C-W, Wong K-C, Chong GS-C, Wong MT-P, Chang SS-Y, et al. A study of psychometric properties, normative scores, and factor structure of the Beck Anxiety Inventory--the Chinese version. *Chinese Journal of Clinical Psychology* (2002).
140. Kroenke K, Spitzer RL, Williams JB. The PHQ-9: validity of a brief depression severity measure. *Journal of general internal medicine* (2001) 16(9):606-13. doi: 10.1046/j.1525-1497.2001.016009606.x.
141. 王春芳, 蔡则环, 徐清. 抑郁自评量表—SDS 对 1,340 例正常人评定分析. *中国神经精神疾病杂志* (1986) 12(5):267-8.
142. Zung WW. A self-rating depression scale. *Archives of general psychiatry* (1965) 12(1):63-70. doi: 10.1001/archpsyc.1965.01720310065008.
143. Cheung YB, Liu KY, Yip PS. Performance of the CES-D and its short forms in screening suicidality and hopelessness in the community. *Suicide and Life-Threatening Behavior* (2007) 37(1):79-88. doi: 10.1521/suli.2007.37.1.79.
144. Zimmerman M, Martinez JH, Young D, Chelminski I, Dalrymple K. Severity classification on the Hamilton depression rating scale. *J Affect Disord* (2013) 150(2):384-8. doi: 10.1016/j.jad.2013.04.028.
145. Wang Z, Yuan C-M, Huang J, Li Z-Z, Chen J, Zhang H-Y, et al. Reliability and validity of the Chinese version of Beck Depression Inventory-II among depression patients. *Chinese Mental Health Journal* (2011).
146. Health WCCiM. Chinese version of the WHO-Five Well-Being Index. (2020) [cited 2020 Feb 10]. Available from: http://www.who-5.org.
147. Kessler RC, Green JG, Gruber MJ, Sampson NA, Bromet E, Cuitan M, et al. Screening for serious mental illness in the general population with the K6 screening scale: results from the WHO World Mental Health (WMH) survey initiative. *International journal of methods in psychiatric research* (2010) 19(S1):4-22. doi: 10.1002/mpr.310.
148. Wu KK, Chan KJSp, epidemiology p. The development of the Chinese version of Impact of Event Scale–Revised (CIES-R). *Social psychiatry and psychiatric epidemiology* (2003) 38(2):94-8. doi: 10.1007/s00127-003-0611-x.
149. Goldberg DP, Hillier VF. A scaled version of the General Health Questionnaire. *Psychological medicine* (1979) 9(1):139-45. doi: 10.1017/S0033291700021644.
150. Wang J, Guo W-j, Zhang L, Deng W, Wang H-y, Yu J-y, et al. The development and validation of Huaxi emotional-distress index (HEI): a Chinese questionnaire for screening depression and anxiety in non-psychiatric clinical settings. *Comprehensive psychiatry* (2017) 76:87-97. doi: 10.1016/j.comppsych.2017.04.001.
151. Morin CM, Belleville G, Bélanger L, Ivers HJS. The Insomnia Severity Index: psychometric indicators to detect insomnia cases and evaluate treatment response. *Sleep* (2011) 34(5):601-8. doi: 10.1093/sleep/34.5.601.

152. Tsai P-S, Wang S-Y, Wang M-Y, Su C-T, Yang T-T, Huang C-J, et al. Psychometric evaluation of the Chinese version of the Pittsburgh Sleep Quality Index (CPSQI) in primary insomnia and control subjects. *Quality of Life Research* (2005) 14(8):1943-52. doi: 10.1007/s11136-005-4346-x.
153. Buysse DJ, Reynolds III CF, Monk TH, Berman SR, Kupfer DJ. The Pittsburgh Sleep Quality Index: a new instrument for psychiatric practice and research. *Psychiatry Res* (1989) 28(2):193-213. doi: 10.1016/0165-1781(89)90047-4.
154. Soldatos CR, Dikeos DG, Paparrigopoulos TJ. Athens Insomnia Scale: validation of an instrument based on ICD-10 criteria. *Journal of psychosomatic research* (2000) 48(6):555-60. doi: 10.1016/S0022-3999(00)00095-7.
155. Lee Y, Rosenblat JD, Lee J, Carmona NE, Subramaniapillai M, Shekotikhina M, et al. Efficacy of antidepressants on measures of workplace functioning in major depressive disorder: A systematic review. *J Affect Disord* (2018) 227:406-15. doi: 10.1016/j.jad.2017.11.003.
156. Guo J, He H, Fu M, Han Z, Qu Z, Wang X, et al. Suicidality associated with PTSD, depression, and disaster recovery status among adult survivors 8 years after the 2008 Wenchuan earthquake in China. *Psychiatry Res* (2017) 253:383-90. doi: 10.1016/j.psychres.2017.04.022.
157. Creamer M, Bell R, Failla S. Psychometric properties of the impact of event scale—revised. *Behaviour Research and Therapy* (2003) 41(12):1489-96. doi: 10.1016/j.brat.2003.07.010.
158. Blevins CA, Weathers FW, Davis MT, Witte TK, Domino JL. The posttraumatic stress disorder checklist for DSM-5 (PCL-5): Development and initial psychometric evaluation. *Journal of traumatic stress* (2015) 28(6):489-98. doi: 10.1002/jts.22059.
159. Dobie DJ, Kivlahan DR, Maynard C, Bush KR, McFall M, Epler AJ, et al. Screening for post-traumatic stress disorder in female Veteran's Affairs patients: validation of the PTSD checklist. *General hospital psychiatry* (2002) 24(6):367-74. doi: 10.1016/S0163-8343(02)00207-4.
160. Wilberforce N, Wilberforce K, Aubrey-Bassler FK. Post-traumatic stress disorder in physicians from an underserviced area. *Family practice* (2010) 27(3):339-43. doi: 10.1093/fampra/cmq002.
161. Schlenger WE, Caddell JM, Ebert L, Jordan BK, Rourke KM, Wilson D, et al. Psychological reactions to terrorist attacks: findings from the National Study of Americans' Reactions to September 11. *JAMA* (2002) 288(5):581-8. doi: 10.1001/jama.288.5.581.
162. Thoresen S, Tambs K, Hussain A, Heir T, Johansen VA, Bisson JIJSp, et al. Brief measure of posttraumatic stress reactions: Impact of Event Scale-6. *Social psychiatry and psychiatric epidemiology* (2010) 45(3):405-12. doi: 10.1007/s00127-009-0073-x.
163. Prins A, Bovin MJ, Smolenski DJ, Marx BP, Kimerling R, Jenkins-Guarnieri MA, et al. The primary care PTSD screen for DSM-5 (PC-PTSD-5): development and evaluation within a veteran primary care sample. *Journal of general internal medicine* (2016) 31(10):1206-11. doi: 10.1007/s11606-016-3703-5.
